# Supplementary material for: A plausible identifiable model of the canonical NF-κB signaling pathway
Source: PLoS One. 2023 Jun 2;18(6):e0286416. doi: 10.1371/journal.pone.0286416 (PMC10237389; doi:10.1371/journal.pone.0286416)
Supplement: S1 Fig — The details for Hoffmann et al. 2002 model simulations are provided in S1 Text. (PDF) [file pone.0286416.s001.pdf]

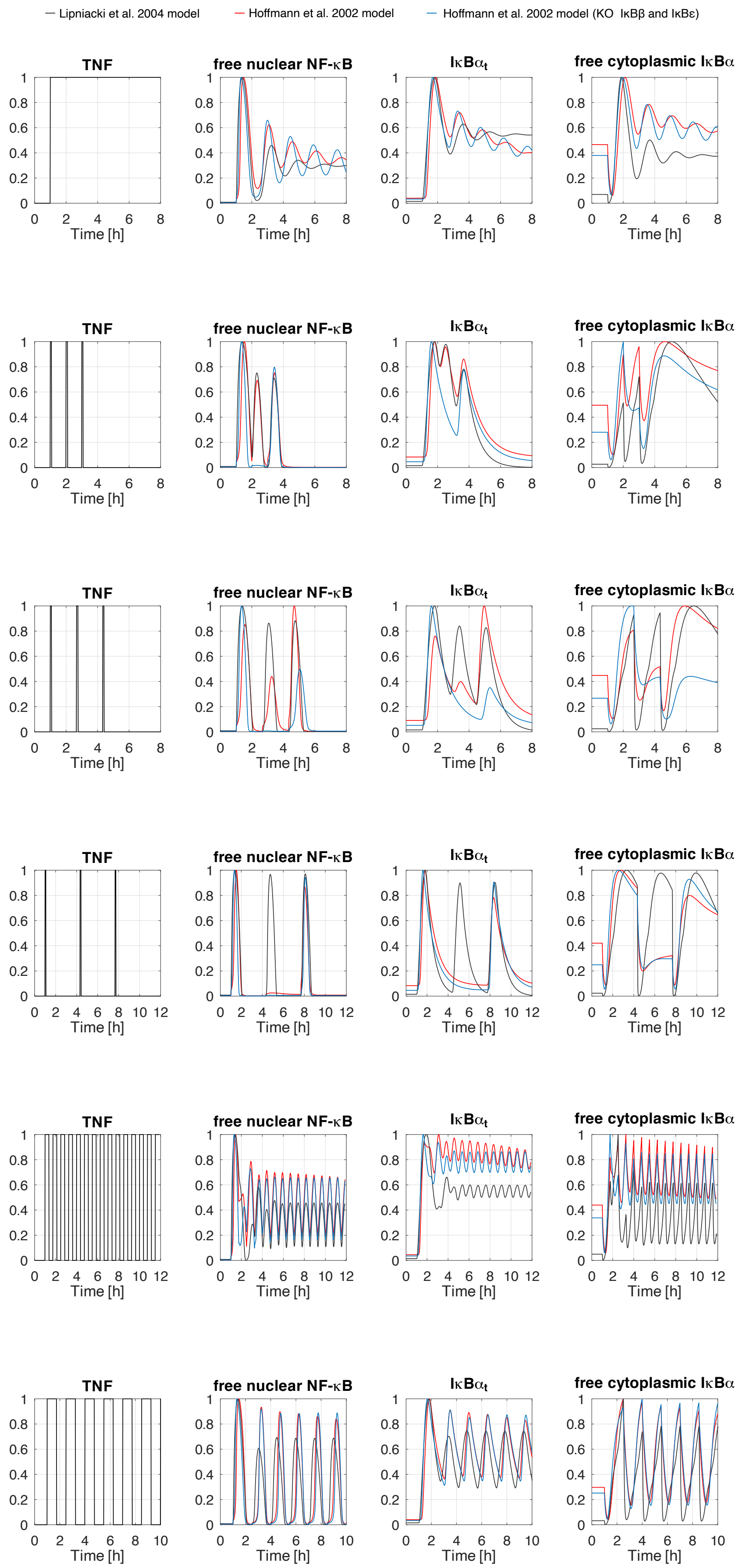

S1 Fig. Comparison between Lipniacki et al. 2004 model and Hoffmann et al. 2002 model in two variants: with and without Iκ$B\beta$ **and Iκ**$B\varepsilon$ **isoforms**. The details for Hoffmann et al. 2002 model simulations are provided in S1 Text.
