# Supplementary material for: A plausible identifiable model of the canonical NF-κB signaling pathway
Source: PLoS One. 2023 Jun 2;18(6):e0286416. doi: 10.1371/journal.pone.0286416 (PMC10237389; doi:10.1371/journal.pone.0286416)
Supplement: S2 Fig — The details for Ashall et al. 2009 model and Murakawa et al. 2015 model simulations are provided in S1 Text. (PDF) [file pone.0286416.s002.pdf]

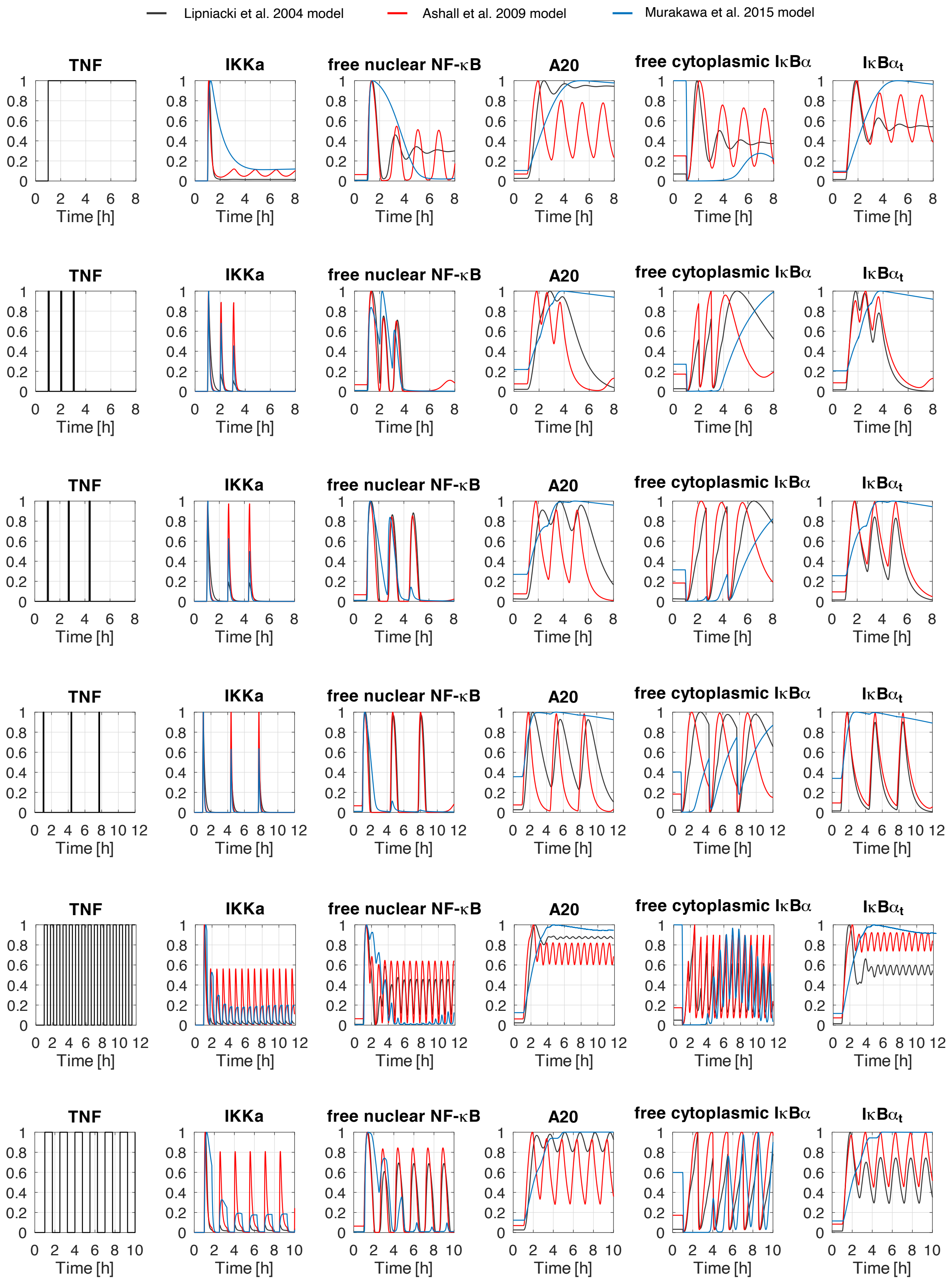

S2 Fig. Comparison between Lipniacki et al. 2004 model, Ashall et al. 2009 model, and Murakawa et al. 2015 model in WT cells. The details for Ashall et al. 2009 model and Murakawa et al. 2015 model simulations are provided in S1 Text.
