# Supplementary material for: A plausible identifiable model of the canonical NF-κB signaling pathway
Source: PLoS One. 2023 Jun 2;18(6):e0286416. doi: 10.1371/journal.pone.0286416 (PMC10237389; doi:10.1371/journal.pone.0286416)
Supplement: S4 Fig — Red dots indicate time points from which the nuclear NF-κB and total IκBα protein in silico measurements are used for fitting the reduced model to the original one. The time points for the remaining variables are given in S1 Table. (PDF) [file pone.0286416.s004.pdf]

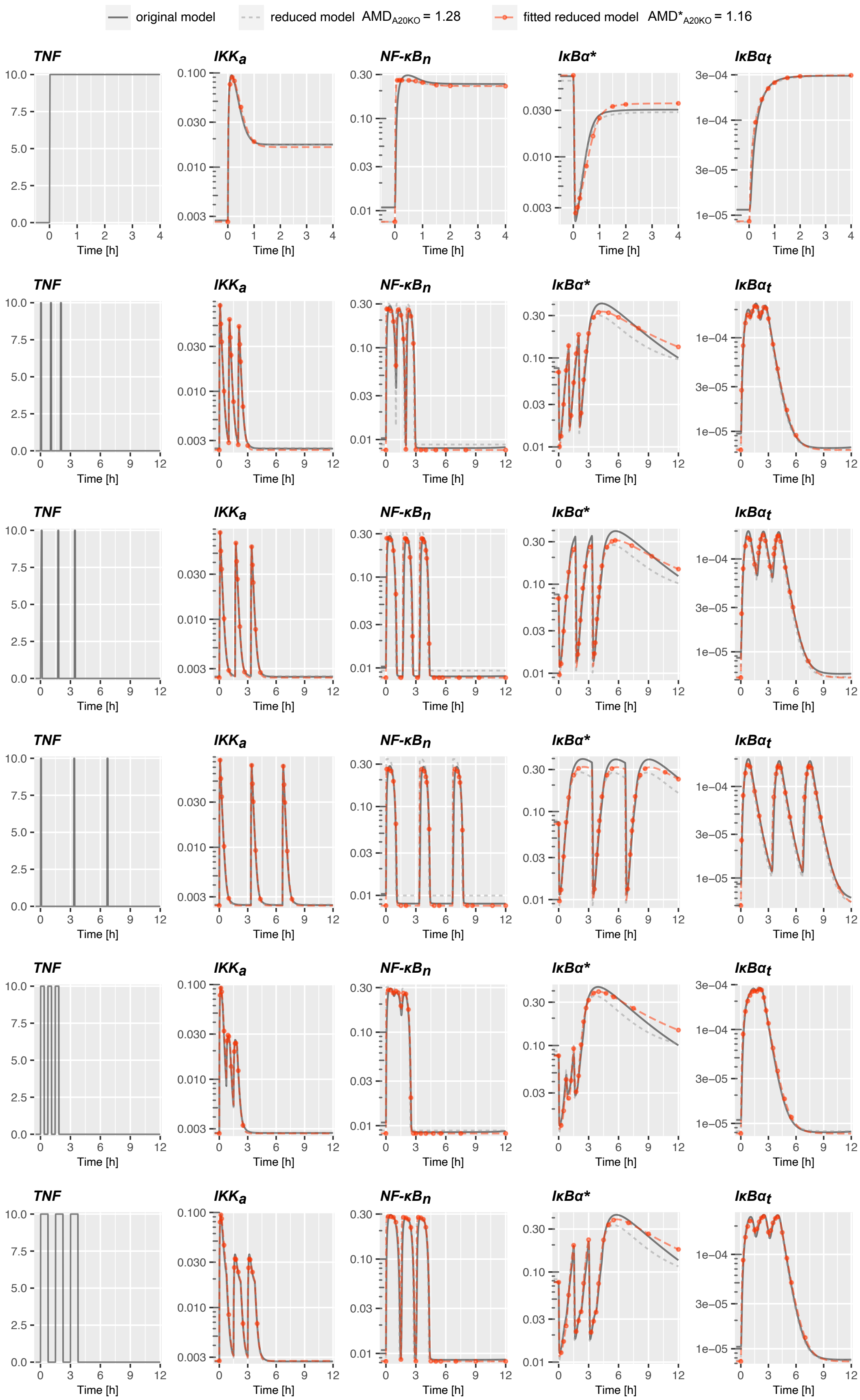

S4 Fig. Dynamics of the original, reduced and reduced fitted models in combination experiment in A20 KO cells defined in S1 Table. Red dots indicate time points from which the nuclear NF-κB and total IκBα protein *in silico* measurements are used for fitting the reduced model to the original one. The time points for the remaining variables are given in S1 Table.
