# Supplementary material for: A plausible identifiable model of the canonical NF-κB signaling pathway
Source: PLoS One. 2023 Jun 2;18(6):e0286416. doi: 10.1371/journal.pone.0286416 (PMC10237389; doi:10.1371/journal.pone.0286416)
Supplement: S5 Fig — Simulations are performed for the combination experiment defined in S1 Table. For each parameter set the corresponding AMDA20KO* is computed based on trajectories of the 5 main model variables. (PDF) [file pone.0286416.s005.pdf]

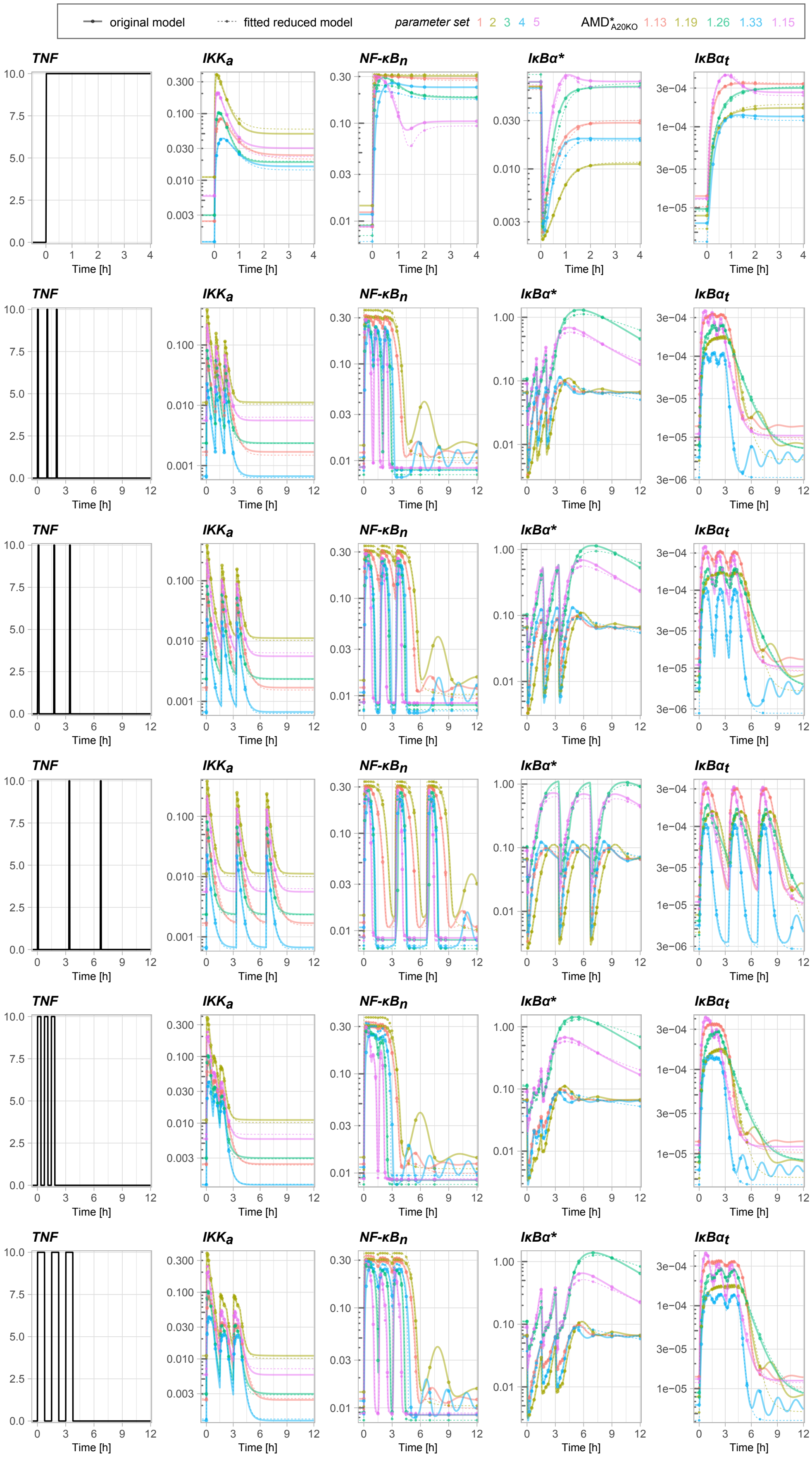


**S5 Fig. Simulations of the original model for A20 KO cells for five different sets of parameters and the corresponding reduced model with refitted parameter values (see S3 Table and S4 Table).** Simulations are performed for the combination experiment defined in S1 Table. For each parameter set the corresponding $AMD^{*}_{A20KO}$ is computed based on trajectories of the 5 main model variables.
