# Supplementary figures and images for: A plausible identifiable model of the canonical NF-κB signaling pathway
Source: PLoS One. 2023 Jun 2;18(6):e0286416. doi: 10.1371/journal.pone.0286416 (PMC10237389; doi:10.1371/journal.pone.0286416)

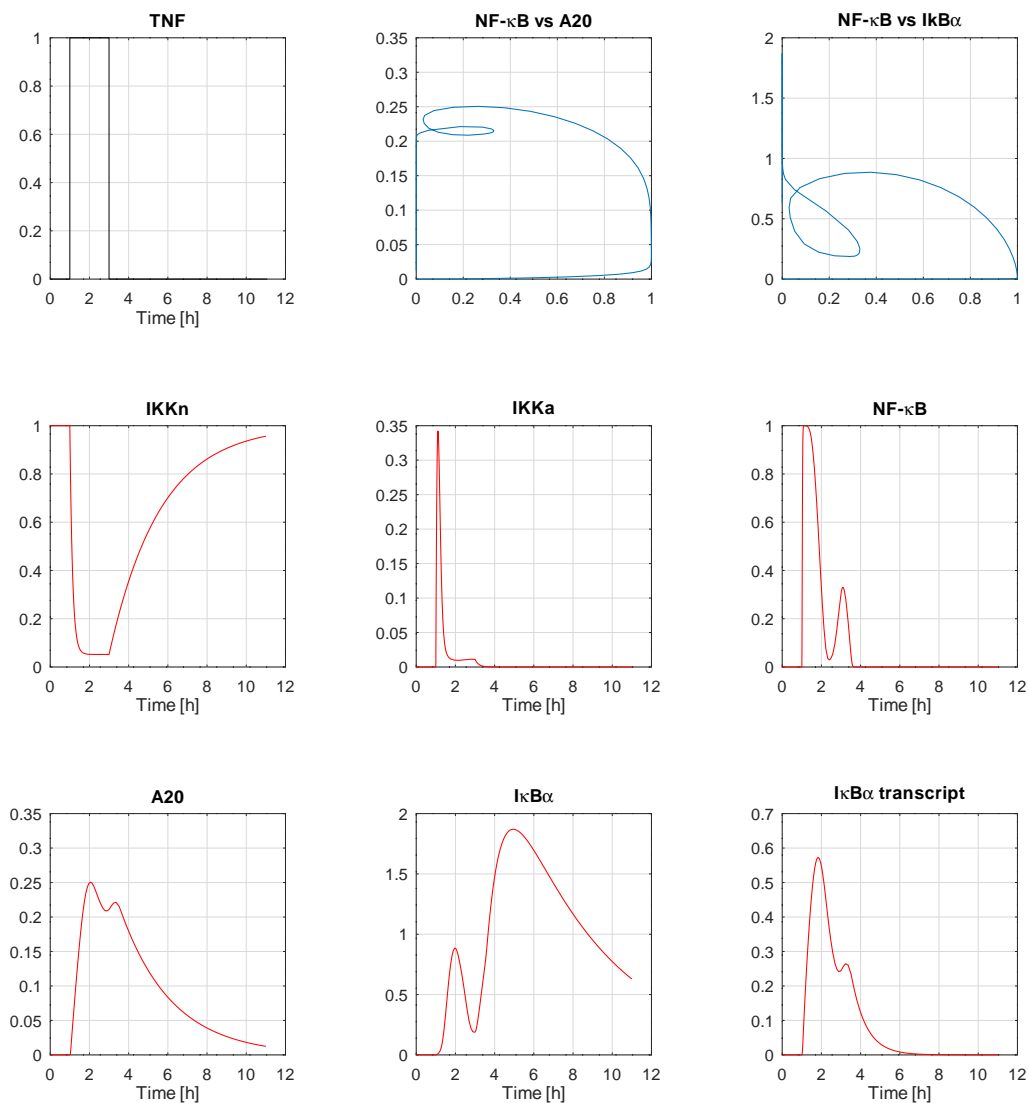

**S6 Fig. Dynamics of the reduced fitted model for the on-off protocol in WT cells defined in Table 2.**

Supplement: S6 Fig — (PDF) [file pone.0286416.s006.pdf]
