# Supplementary material for: A plausible identifiable model of the canonical NF-κB signaling pathway
Source: PLoS One. 2023 Jun 2;18(6):e0286416. doi: 10.1371/journal.pone.0286416 (PMC10237389; doi:10.1371/journal.pone.0286416)
Supplement: S7 Fig — A comparison of 75% confidence ellipses (shown in black) computed for σdata = 1.3 in the linear sensitivity matrix based analysis with results from 50 Monte Carlo simulations for four values of σdata (1.0, 1.1, 1.2, 1.3). Shown are projections on 21 planes spanned by 7 parameters with the smallest geometric standard deviations σcarlo,j (for σdata = 1.3). (PDF) [file pone.0286416.s007.pdf]

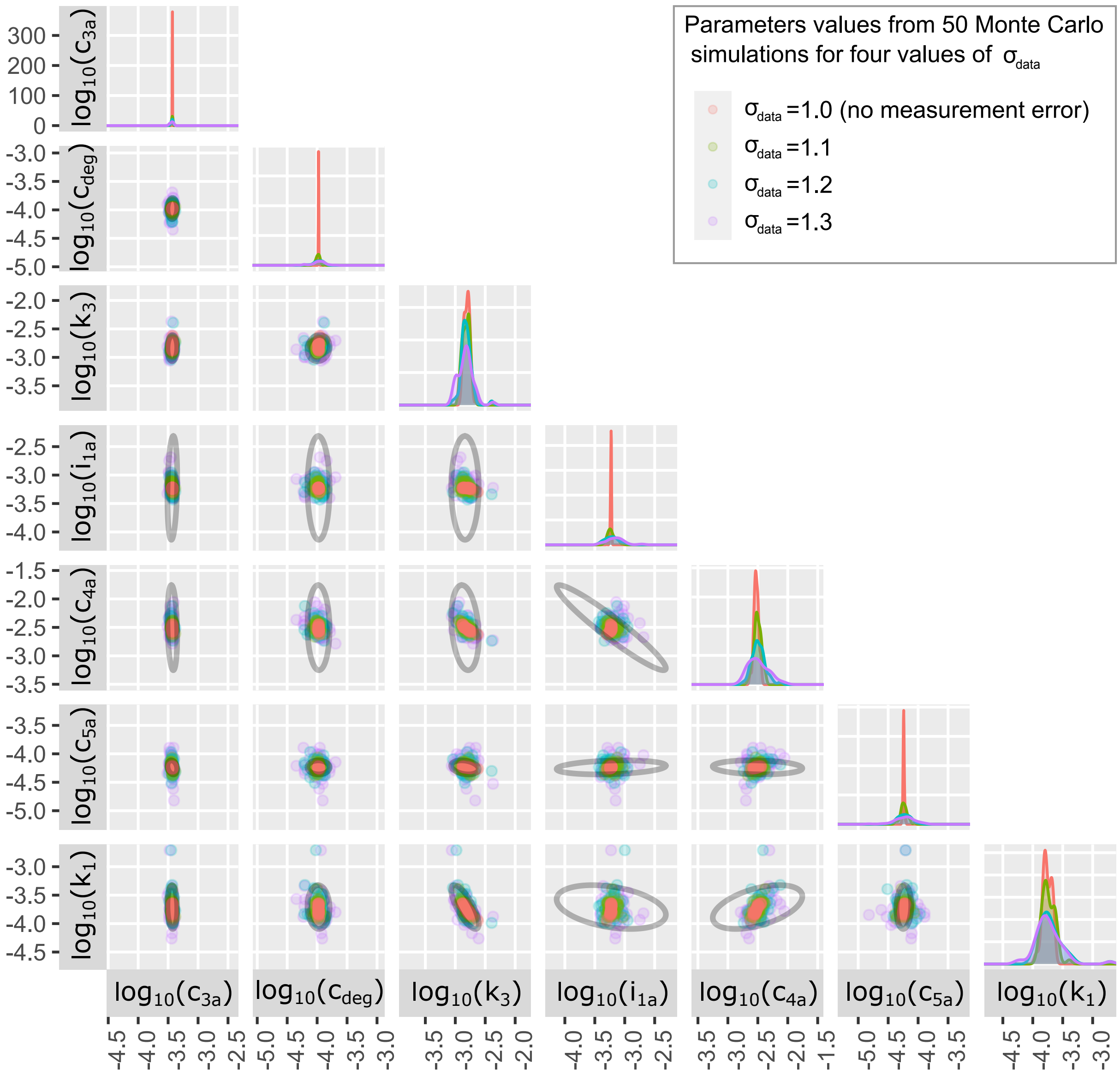

S7 Fig. Practical identifiability of the reduced model based on Monte Carlo simulations. A comparison of 75% confidence ellipses (shown in black) computed for $\sigma_{data} = 1.3$ in the linear sensitivity matrix based analysis with results from 50 Monte Carlo simulations for four values of σ_{data} (1.0, 1.1, 1.2, 1.3). Shown are projections on 21 planes spanned by 7 parameters with the smallest geometric standard deviations $\sigma_{carlo,j}$ (for $\sigma_{data} = 1.3$).
