# Supplementary material for: A plausible identifiable model of the canonical NF-κB signaling pathway
Source: PLoS One. 2023 Jun 2;18(6):e0286416. doi: 10.1371/journal.pone.0286416 (PMC10237389; doi:10.1371/journal.pone.0286416)
Supplement: S8 Fig — In contrast to the original Krishna et al. assumption, it is assumed that the parameter C (governing IκBα degradation) remains constant, while the parameter A (multiplicating the source term for NF-κB) attains its nominal value, when TNF is on, and zero value, when TNF is off. The trajectories are plotted in log scale; dots denote fitting time points, and the trajectories are plotted as saw lines between these points. The discrepancy between free cytoplasmic IκBα trajectories causes that average multiplicative distance between these two models equals 2.06. (PDF) [file pone.0286416.s008.pdf]

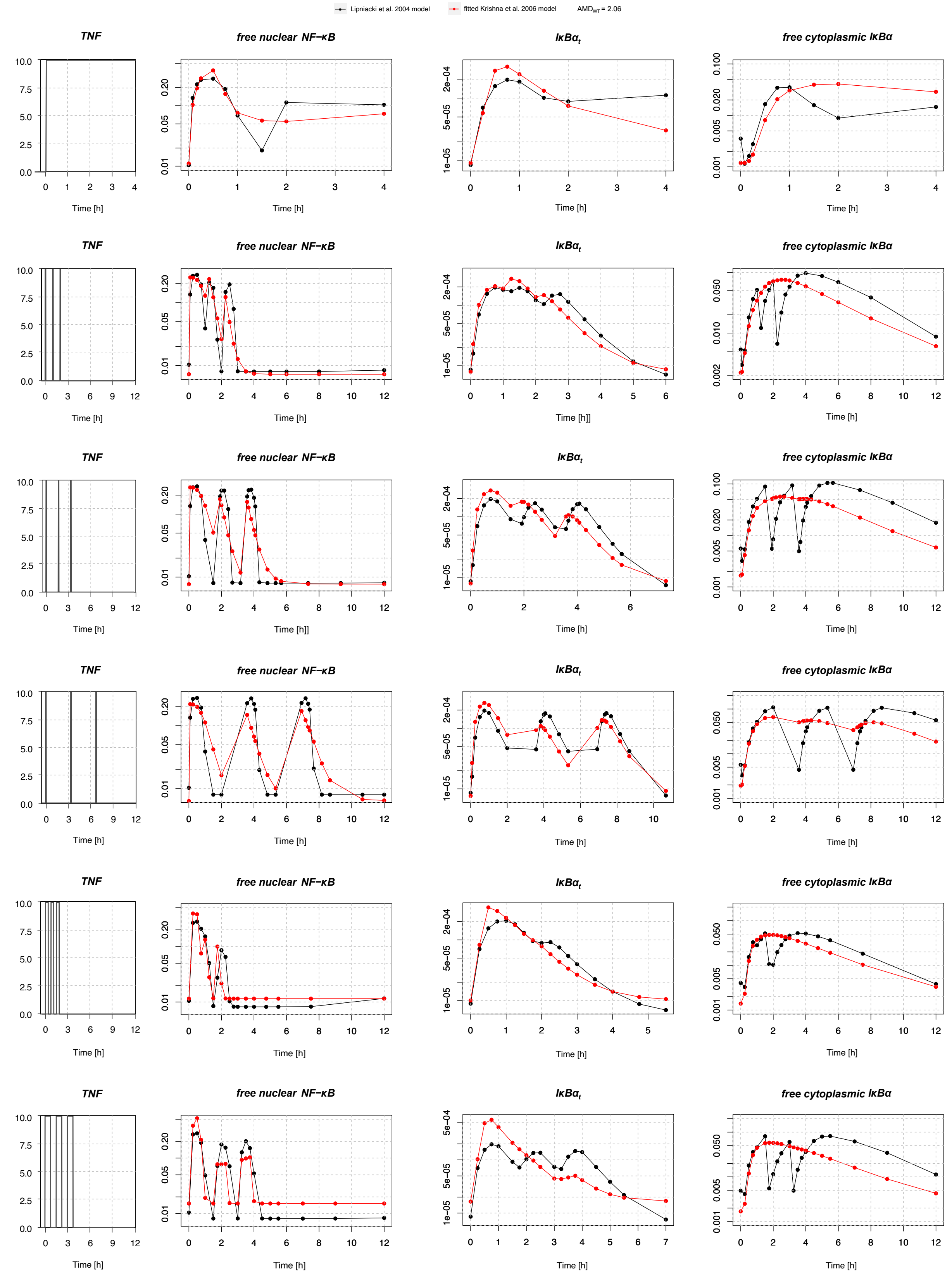

S8 Fig. Fit of 'reinterpreted' Krishna et al. 2006 model to Lipniacki et al. 2004 model. In contrast to the original Krishna et al. assumption, it is assumed that the parameter C (governing IκBα degradation) remains constant, while the parameter A (multiplicating the source term for NF-κB) attains its nominal value, when TNF is on, and zero value, when TNF is off. The trajectories are plotted in log scale; dots denote fitting time points, and the trajectories are plotted as saw lines between these points. The discrepancy between free cytoplasmic IκBα trajectories causes that average multiplicative distance between these two models equals 2.06.
