# Supplementary material for: A plausible identifiable model of the canonical NF-κB signaling pathway
Source: PLoS One. 2023 Jun 2;18(6):e0286416. doi: 10.1371/journal.pone.0286416 (PMC10237389; doi:10.1371/journal.pone.0286416)
Supplement: S1 Table — Each table block describes one TNF stimulation protocol for which the measured variables are: IKKa, nuclear NF-κB, A20, total IκBα protein denoted by IκBα* (IκBα* = IκBα + 1 − NFκB), and IκBα mRNA. For each variable the measurement times used for the sensitivity-based identifiability analysis and reduced model fitting are given. (PDF) [file pone.0286416.s009.pdf]

S1 Table. Summary of the considered stimulation protocols of the NF-κB system in WT and A20 KO cells used for *in silico* experiments. Each table block describes one TNF stimulation protocol for which the measured variables are: IKKa, nuclear NF-κB, A20, total IκBα protein denoted by IκBα^* ($I\kappa B\alpha^* = I\kappa B\alpha + 1 - NF\kappa B$) , and I$\kappa$B$\alpha$ mRNA. For each variable the measurement times used for the sensitivity-based identifiability analysis and reduced model fitting are given.

**continuous**	**TNF *ON* 0-240min**	Reference: [1]
IKKa	0, 5, 10, 15, 30, 60 min	
NF-κB, IκBα^*	0, 5, 10, 15, 30, 45, 60, 90, 120, 240 min	
A20, IκBα_t	0, 15, 30, 45, 60, 90, 120, 240 min	
**pulse 5-60**	**TNF *ON* 0-5 min, 60-65 min, 120-125 min**	Reference: [1]
IKKa	0,5, 10, 15, 30, 60, 65, 70, 75, 90, 120, 125, 130, 135, 150, 180 min	
NF-κB, IκBα^*	0,5, 15, 30, 45, 60, 75, 90, 105, 120, 135, 150, 165, 180, 210, 240, 300, 360, 480, 720 min	
A20, IκBα_t	0,5, 15, 30, 45, 60, 75, 90, 105, 120, 135, 150, 165, 180, 210, 240, 300, 360 min	
**pulse 5-100**	**TNF *ON* 0-5 min, 100-105 min, 200-205 min**	Reference: [1]
IKKa	0, 5, 10, 15, 30, 60, 105, 110, 115, 130, 160, 205, 210, 215, 230, 260 min	
NF-κB, IκBα^*	0, 5, 15, 30, 45, 60, 90, 115, 120, 130, 145, 160, 190, 215, 220, 230, 240, 245, 260, 290, 320, 340, 440, 560, 720 min	
A20, IκBα_t	0, 5, 15, 30, 45, 60, 90, 115, 120, 130, 145, 160, 190, 215, 220, 230, 240, 245, 260, 290, 320, 340, 440 min	
**pulse 5-200**	**TNF *ON* 0-5 min, 200-205 min, 400-405 min**	Reference:[1]
IKKa	0, 5, 10, 15, 30, 60, 205, 210, 215, 230, 260, 405, 410, 415, 430, 460 min	
NF-κB, IκBα^*	0, 5, 15, 30, 45, 60, 90, 120, 215, 230, 240, 245, 260, 290, 320, 415, 430, 440, 445, 460, 490, 520, 640, 720 min	
A20, IκBα_t	0, 5, 15, 30, 45, 60, 90, 120, 215, 230, 240, 245, 260, 290, 320, 415, 430, 440, 445, 460, 490, 520, 640 min	
**pulse 22.5-45**	**TNF *ON* 0-22.5 min, 45-67.5 min, 90-112.5 min**	Reference: [2]
IKKa	0, 5, 10, 15, 30, 50, 55, 60, 75, 95, 100, 105, 120, 150 min	
NF-κB, IκBα^*	0, 15 30, 45, 60, 75, 90, 105, 120, 135, 150, 165, 180, 210, 240, 285, 330, 450, 720 min	
A20, IκBα_t	0, 15 30, 45, 60, 75, 90, 105, 120, 135, 150, 165, 180, 210, 240, 285, 330 min	
**pulse 45-90**	**TNF *ON* 0-45 min, 90-135 min, 180-225 min**	Reference: [2]
IKKa	0, 5, 10, 15, 30, 60, 95, 100, 105, 120, 150, 185, 190, 195, 210, 240 min	
NF-κB, IκBα^*	0, 15, 30, 45, 60, 90, 105, 120, 135, 150, 180, 195, 210, 225, 240, 270, 300, 330, 420, 540, 720 min	
A20, IκBα_t	0, 15, 30, 45, 60, 90, 105, 120, 135, 150, 180, 195, 210, 225, 240, 270, 300, 330, 420 min	

[1] Ashall L, et al. Pulsatile stimulation determines timing and specificity of NF-κB-dependent transcription. Science. 2009; 324: 242–246. doi: 10.1126/science.1164860. PMID: 19359585

[2] Zambrano S, De Toma I, Piffer A, Bianchi ME, Agresti A. NF-κB oscillations translate into functionally related patterns of gene expression. eLife 5:e09100. doi:10.7554/eLife.09100
