# Supplementary material for: A plausible identifiable model of the canonical NF-κB signaling pathway
Source: PLoS One. 2023 Jun 2;18(6):e0286416. doi: 10.1371/journal.pone.0286416 (PMC10237389; doi:10.1371/journal.pone.0286416)
Supplement: S2 Table — Protocol sets are joint for WT and A20 KO. The combination experiment consists of 6 protocols: continuous and all pulsatile ones. The scaling factor is dim. (PDF) [file pone.0286416.s010.pdf]

S2 Table. List of protocol sets, the corresponding numbers of measurement time points (N), and sensitivity vectors dimensions (dim). Protocol sets are joint for WT and A20 KO. The combination experiment consists of 6 protocols: continuous and all pulsatile ones. The scaling factor is $dim^{0.5}$.

**Protocol sets**	**N**	**dim**	**dim**$^{0.5}$
continuous	76	67	8.19
pulse 5-60	166	157	12.53
pulse 5-100	201	192	13.86
pulse 5-200	197	188	13.71
pulse 22.5-45	155	146	12.08
pulse 45-90	173	164	12.81
continuous<br>pulse 5-60	242	224	14.97
continuous<br>pulse 5-100	277	259	16.09
continuous<br>pulse 5-200	273	255	15.97
continuous<br>pulse 22.5-45	231	213	14.59
continuous<br>pulse 45-90	249	231	15.20
combination experiment	968	914	30.23
on-off	50	41	6.40
