# Supplementary material for: A plausible identifiable model of the canonical NF-κB signaling pathway
Source: PLoS One. 2023 Jun 2;18(6):e0286416. doi: 10.1371/journal.pone.0286416 (PMC10237389; doi:10.1371/journal.pone.0286416)
Supplement: S3 Table — Five parameter sets of the original model randomly selected by varying the original parameters at most three-fold from their original values. (PDF) [file pone.0286416.s011.pdf]

S3 Table. Parameter sets of the original model. Five parameter sets of the original model randomly selected by varying the original parameters at most three-fold from their original values.

Parameter	Set 1	Set 2	Set 3	Set 4	Set 5
c_1	6.74E-07	7.04E-07	1.85E-07	3.31E-07	5.61E-07
c_3	9.13E-04	1.96E-04	7.11E-04	1.92E-04	3.04E-04
c_4	4.71E-01	2.45E-01	8.66E-01	1.33E+00	3.06E-01
c_5	4.88E-04	1.30E-04	8.78E-04	5.73E-04	2.75E-04
k_1	2.04E-03	5.85E-03	2.11E-03	8.48E-04	4.65E-03
k_2	1.01E-01	3.90E-02	8.93E-02	2.57E-01	2.94E-01
k_3	5.60E-04	8.46E-04	9.50E-04	7.88E-04	7.92E-04
k_{prod}	1.50E-05	3.57E-05	1.70E-05	1.63E-05	2.16E-05
k_{deg}	1.10E-04	6.74E-05	8.61E-05	1.44E-04	7.48E-05
a_2	1.87E-01	2.82E-01	1.26E-01	1.11E-01	1.32E-01
a_1	8.33E-01	4.67E-01	5.35E-01	9.84E-01	9.70E-01
a_3	8.00E-01	2.54E+00	1.33E+00	1.33E+00	3.34E-01
t_1	6.83E-02	2.02E-01	4.43E-02	1.89E-01	2.14E-01
t_2	2.42E-01	4.88E-02	1.36E-01	2.12E-01	1.33E-01
c_{1a}	1.01E-06	2.76E-07	3.49E-07	5.39E-07	1.13E-06
c_{3a}	8.63E-04	4.91E-04	2.07E-04	9.36E-04	4.29E-04
c_{4a}	3.83E-01	5.47E-01	1.13E+00	9.67E-01	1.42E+00
c_{5a}	2.69E-04	2.91E-04	1.12E-04	1.06E-04	1.05E-04
c_{6a}	1.33E-05	1.11E-05	1.14E-05	3.74E-05	7.45E-06
i_1	4.65E-03	6.45E-03	2.57E-03	9.69E-04	5.32E-03
e_{2a}	3.48E-03	3.67E-03	4.53E-03	1.03E-02	9.59E-03
i_{1a}	3.76E-04	4.67E-04	6.66E-04	7.86E-04	1.24E-03
e_{1a}	5.62E-04	1.07E-03	2.16E-04	4.78E-04	4.68E-04
