# Supplementary material for: A plausible identifiable model of the canonical NF-κB signaling pathway
Source: PLoS One. 2023 Jun 2;18(6):e0286416. doi: 10.1371/journal.pone.0286416 (PMC10237389; doi:10.1371/journal.pone.0286416)
Supplement: S4 Table — (PDF) [file pone.0286416.s012.pdf]

S4 Table. Fitted parameter sets of the reduced model obtained from fitting to the original model with varied parameter sets.

Parameter	Fit 1	Fit 2	Fit 3	Fit 4	Fit 5
a_2	2.43E-02	1.19E-01	4.69E-02	2.62E-02	1.65E-02
a_3	7.10E-02	4.04E-01	1.71E-01	5.03E-02	9.17E-02
c_{4a}	2.57E-03	2.18E-03	7.94E-03	2.78E-03	1.35E-02
δ	7.92E-02	5.32E-01	3.92E-02	3.86E-02	8.55E-03
ε	2.96E-02	1.67E-01	7.51E-02	1.67E-03	2.83E-02
k_2	1.59E-02	1.28E-01	6.25E-03	8.55E-02	1.29E-01
c_{3a}	7.44E-04	4.00E-04	1.94E-04	7.61E-04	3.50E-04
c_{5a}	4.24E-04	1.00E-03	4.04E-05	4.19E-05	6.04E-05
c_{deg}	2.56E-04	1.71E-05	3.02E-04	6.89E-05	6.82E-05
i_{1a}	3.62E-04	7.30E-04	4.59E-04	3.56E-04	6.82E-04
k_1	1.84E-03	9.44E-03	2.12E-03	9.37E-04	4.97E-03
k_3	4.72E-04	7.25E-04	9.30E-04	7.20E-04	8.11E-04
k_{deg}	8.04E-05	8.33E-05	9.02E-05	1.20E-04	4.81E-05
