# Supplementary material for: A plausible identifiable model of the canonical NF-κB signaling pathway
Source: PLoS One. 2023 Jun 2;18(6):e0286416. doi: 10.1371/journal.pone.0286416 (PMC10237389; doi:10.1371/journal.pone.0286416)
Supplement: S1 Text — (PDF) [file pone.0286416.s013.pdf]

S1 Text

Details of NF-κB models simulations

featuring the article

A plausible identifiable model of the canonical NF-κB signaling pathway

by Joanna Jaruszewicz-Błońska, Ilona Kosiuk, Wiktor Prus, and Tomasz Lipniacki

all at the Institute of Fundamental Technological Research

of the Polish Academy of Sciences, Warsaw, Poland

This supplementary material documents numerical simulations of existing computational models of the NF-κB signaling pathway: Hoffmann et al. 2002 model [1], Ashall et al. 2009 model [2], Murakawa et al. 2015 model [3], and Krishna et al. 2006 model [4].

We used the encoded model "BIOMD0000000140" from BioModels Database[5] to simulate the Hoffmann et al. 2002 model. The encoded model has been modified to run both continuous and pulsatile TNF protocols. The code for the Ashall et al. 2009 model provided by the authors has been adjusted to run both continuous and pulsatile TNF protocols. We encoded the Murakawa et al. 2015 model based on the equations and protocol in WT cells described in the original work[3]. We encoded the Krishna et al. 2006 model and modified it for the studied pulsatile protocols.

[1] Hoffmann A, Levchenko A, Scott ML, Baltimore D. The IkappaB-NF-kappaB signaling module: temporal control and selective gene activation. Science. 2002 Nov 8;298(5596): 1241-5.

[2] Ashall L, Horton CA, Nelson DE, Paszek P, Harper CV, Sillitoe K, et al. Pulsatile stimulation determines timing and specificity of NF-kappaB-dependent transcription. Science. 2009;324(5924): 242-6.

[3] Murakawa Y, Hinz M, Mothes J, Schuetz A, Uhl M, Wyler E, et al. RC3H1 post-transcriptionally regulates A20 mRNA and modulates the activity of the IKK/NF-kappaB pathway. Nat. Commun. 2015 Jul 14;6(1).

[4] Krishna S, Jensen M, Sneppen K. Minimal model of spiky oscillations in NF-κB signalling. PNAS. 2006; 103(29): 10840-10845.

[5] Li C, Donizelli M, Rodriguez N, Dharuri H, Endler L, Chelliah V, et al. BioModels Database: An enhanced, curated and annotated resource for published quantitative kinetic models. BMC Syst Biol. 2010;4(1): 92.

1 Hoffmann et al. 2002 model

The model contains 27 variables, i.e., 24 species and 3 auxiliary variables (used to model changing fraction of IκBβ and IκBβ - NF-κB complexes that may be translocated from the nucleus), see Table 1.

Table 1: **Model variables and initial conditions.**

Variable	Name	Compartment	Int.condition
x(1)	IkBalpha	cytoplasm	x0(1) = 0.0
x(2)	NFkB	cytoplasm	x0(2) = 0.0
x(3)	IkBalpha_NFkB	cytoplasm	x0(3) = 0.1
x(4)	IkBbeta	cytoplasm	x0(4) = 0.0
x(5)	IkBbeta_NfkB	cytoplasm	x0(5) = 0.0
x(6)	IkBeps	cytoplasm	x0(6) = 0.0
x(7)	IkBeps_NFkB	cytoplasm	x0(7) = 0.0
x(8)	IKK_IkBalpha	cytoplasm	x0(8) = 0.0
x(9)	IKK_IkBalpha_NfkB	cytoplasm	x0(9) = 0.0
x(10)	IKK	cytoplasm	x0(10) = 0.0
x(11)	IKK_IkBbeta	cytoplasm	x0(11) = 0.0
x(12)	IKK_IkBbeta_NfkB	cytoplasm	x0(12) = 0.0
x(13)	IKK_IkBeps	cytoplasm	x0(13) = 0.0
x(14)	IKK_IkBeps_NFkB	cytoplasm	x0(14) = 0.0
x(15)	NFkB_nuc	nucleus	x0(15) = 0
x(16)	IkBalpha_nuc	nucleus	x0(16) = 0.0
x(17)	IkBalpha_nuc_NFkB_nuc	nucleus	x0(17) = 0.0
x(18)	IkBbeta_nuc	nucleus	x0(18) = 0.0
x(19)	IkBbeta_nuc_NfkB_nuc	nucleus	x0(19) = 0.0
x(20)	IkBeps_nuc	nucleus	x0(20) = 0.0
x(21)	IkBalpha_transcript	nucleus	x0(21) = 0.0
x(22)	IkBbeta_transcript	nucleus	x0(22) = 0.0
x(23)	IkBeps_transcript	nucleus	x0(23) = 0.0
x(24)	IkBeps_nuc_NfkB_nuc	nucleus	x0(24) = 0.0
x(25)	auxiliary variable		x0(25) = 0.0
x(26)	auxiliary variable		x0(26) = 0.0
x(27)	auxiliary variable		x0(27) = 1.0

The Hoffmann et al. 2002 model contains 45 reactions described in Table 2.

Table 2: **Reactions in the Hoffmann et al. 2002 model.**

id	Reaction
v1	NFkB-IkBalpha complex formation
v2	NFkB-IkBbeta complex formation
v3	NFkB-IkBeps complex formation
v4	NFkB-binary IKK IkBalpha complex formation
v5	IkBalpha degradation
v6	NFkB binary IKK IkBbeta complex formation
v7	IkBbeta degradation
v8	NFkB binary IKK IkBeps complex formation
v9	IkBeps degradation
v10	IkBalpha degradation
v11	IkBbeta degradation
v12	IkBeps degradation
v13	NFkB translocation
v14	NFkB-IkBalpha complex formation
v15	NFkB-IkBbeta complex formation
v16	NFkB-IkBeps complex formation
v17	IkBalpha transcription
v18	IkBalpha inducible transcription
v19	IkBalpha transcript degradation
v20	IkBbeta transcription
v21	IkBbeta transcript degradation
v22	IkBeps transcription
v23	IkBeps transcript degradation
v24	IKK-IkBalpha complex formation
v25	IkBalpha synthesis
v26	IkBalpha degradation
v27	IkBalpha translocation
v28	IKK-IkBbeta complex formation
v29	IkBbeta synthesis
v30	IkBbeta degradation
v31	IkBbeta translocation
v32	IKK-IkBeps complex formation
v33	IkBeps synthesis
v34	IkBeps degradation
v35	IkBeps translocation
v36	IKK-binary IkBalpha NFkB complex formation
v37	IkBalpha NFkB translocation
v38	IKK binary IkBbeta NFkB complex formation
v39	IkBbeta NFkB translocation
v40	IKK binary IkBeps NFkB complex formation
v41	IkBeps NFkB translocation
v42	IkBalpha degradation
v43	IkBbeta degradation
v44	IkBeps degradation
v45	IKK consumption

The corresponding kinetics laws are given by:

$$
\begin{aligned}
v1 &= k_{cyt} * (a4 * x(1) * x(2) - d4 * x(3)) \\
v2 &= k_{cyt} * (a5 * x(4) * x(2) - d5 * x(5)) \\
v3 &= k_{cyt} * (a6 * x(6) * x(2) - d6 * x(7)) \\
v4 &= k_{cyt} * (a4 * x(8) * x(2) - d4 * x(9)) \\
v5 &= k_{cyt} * r4 * x(9) \\
v6 &= k_{cyt} * (a5 * x(11) * x(2) - d5 * x(12)) \\
v7 &= k_{cyt} * r5 * x(12) \\
v8 &= k_{cyt} * (a6 * x(13) * x(2) - d6 * x(14)) \\
v9 &= k_{cyt} * r6 * x(14) \\
v10 &= k_{cyt} * deg4 * x(3) \\
v11 &= k_{cyt} * deg4 * x(5) \\
v12 &= k_{cyt} * deg4 * x(7) \\
v13 &= k_{cyt} * k1 * x(2) - k_{nuc} * k01 * x(15) \\
v14 &= k_{nuc} * (a4 * x(16) * x(15) - d4 * x(17)) \\
v15 &= k_{nuc} * (a5 * x(18) * x(15) - d5 * x(19)) \\
v16 &= k_{nuc} * (a6 * x(20) * x(15) - d6 * x(24)) \\
v17 &= k_{nuc} * tr2a \\
v18 &= k_{nuc} * tr2 * x(15)^2 \\
v19 &= k_{nuc} * tr3 * x(21) \\
v20 &= k_{nuc} * tr2b \\
v21 &= k_{nuc} * tr3 * x(22) \\
v22 &= k_{nuc} * tr2e \\
v23 &= k_{nuc} * tr3 * x(23) \\
v24 &= k_{cyt} * (a1 * x(1) * x(10) - d1 * x(8)) \\
v25 &= k_{nuc} * tr1 * x(21) \\
v26 &= k_{cyt} * deg1 * x(1) \\
v27 &= k_{cyt} * tp1 * x(1) - k_{nuc} * tp2 * x(16) \\
v28 &= k_{cyt} * (a2 * x(4) * x(10) - d2 * x(11)) \\
v29 &= k_{nuc} * tr1 * x(22) \\
v30 &= k_{cyt} * deg1 * x(4) \\
v31 &= k_{cyt} * 0.5 * tp1 * x(4) - k_{nuc} * 0.5 * tp2 * x(27) * x(18) \\
v32 &= k_{cyt} * (a3 * x(6) * x(10) - d3 * x(13)) \\
v33 &= k_{nuc} * tr1 * x(23) \\
v34 &= k_{cyt} * deg1 * x(6) \\
v35 &= k_{cyt} * 0.5 * tp1 * x(6) - k_{nuc} * 0.5 * tp2 * x(20) \\
v36 &= k_{cyt} * (a7 * x(10) * x(3) - d1 * x(9)) \\
v37 &= k_{nuc} * k2 * x(17) \\
v38 &= k_{cyt} * (a8 * x(10) * x(5) - d2 * x(12)) \\
v39 &= k_{nuc} * 0.5 * k2 * x(27) * x(19) \\
v40 &= k_{cyt} * (a9 * x(10) * x(7) - d3 * x(14)) \\
v41 &= k_{nuc} * 0.5 * k2eps * x(24) \\
v42 &= k_{cyt} * r1 * x(8) \\
v43 &= k_{cyt} * r2 * x(11) \\
v44 &= k_{cyt} * r3 * x(13) \\
v45 &= k_{cyt} * k02 * x(10).
\end{aligned}
$$

The dynamics of the Hoffmann et al. 2002 model is governed by the following equations:

$$
\begin{aligned}
\text{IkBalpha} \quad \dot{x}(1) &= v25 - v1 - v24 - v26 - v27 && (1)\\
\text{NFkB} \quad \dot{x}(2) &= v5 + v7 + v9 + v10 + v11 + v12 && (2)\\
&\quad -v1 - v2 - v3 - v4 - v6 - v8 - v13 &&\\
\text{IkBalpha\_NFkB} \quad \dot{x}(3) &= v1 + v37 - v10 - v36 && (3)\\
\text{IkBbeta} \quad \dot{x}(4) &= v29 - v2 - v28 - v30 - v31 && (4)\\
\text{IkBbeta\_NfkB} \quad \dot{x}(5) &= v2 + v39 - v11 - v38 && (5)\\
I\text{kBeps} \quad \dot{x}(6) &= v33 - v3 - v32 - v34 - v35 && (6)\\
\text{IkBeps\_NFkB} \quad \dot{x}(7) &= v3 + v41 - v12 - v40 && (7)\\
\text{IKK\_IkBalpha} \quad \dot{x}(8) &= v24 - v4 - v42 && (8)\\
\text{IKK\_IkBalpha\_NfkB} \quad \dot{x}(9) &= v4 + v36 - v5 && (9)\\
I\text{KK} \quad \dot{x}(10) &= v5 + v7 + v9 + v42 + v43 + v44 && (10)\\
&\quad -v24 - v28 - v32 - v36 - v38 - v40 - v45 &&\\
\text{IKK\_IkBbeta} \quad \dot{x}(11) &= v28 - v6 - v43 && (11)\\
\text{IKK\_IkBbeta\_NfkB} \quad \dot{x}(12) &= v6 + v38 - v7 && (12)\\
\text{IKK\_IkBeps} \quad \dot{x}(13) &= v32 - v8 - v44 && (13)\\
\text{IKK\_IkBeps\_NFkB} \quad \dot{x}(14) &= v8 + v40 - v9 && (14)\\
\text{NFkB\_nuc} \quad \dot{x}(15) &= v13 - v14 - v15 - v16 && (15)\\
\text{IkBalpha\_nuc} \quad \dot{x}(16) &= v27 - v14 && (16)\\
\text{IkBalpha\_nuc\_NFkB\_nuc} \quad \dot{x}(17) &= v14 - v37 && (17)\\
\text{IkBbeta\_nuc} \quad \dot{x}(18) &= v31 - v15 && (18)\\
\text{IkBbeta\_nuc\_NfkB\_nuc} \quad \dot{x}(19) &= v15 - v39 && (19)\\
\text{IkBeps\_nuc} \quad \dot{x}(20) &= v35 - v16 && (20)\\
\text{IkBalpha\_transcript} \quad \dot{x}(21) &= v17 + v18 - v19 && (21)\\
\text{IkBbeta\_transcript} \quad \dot{x}(22) &= v20 - v21 && (22)\\
\text{IkBeps\_transcript} \quad \dot{x}(23) &= v22 - v23 && (23)\\
\text{IkBeps\_nuc\_NfkB\_nuc} \quad \dot{x}(24) &= v16 - v41 && (24)\\
\dot{x}(25) &= 0 && (25)\\
\dot{x}(26) &= x(25) && (26)\\
\dot{x}(27) &= -x(25)/(1 + x(26))^2 && (27)
\end{aligned}
$$

The parameter values used for numerical simulations, given in Table 3, are taken from Supplementary Material[1] (file Hoffmann_somrev.pdf) and expressed in min, except the parameter k02. The value of k02 is equal to the value given in the file Hoffmann_som.pdf, i.e., k02_tnf_on=0.00498 $\text{min}^{-}1$ (half-life of 2.3 hrs), and for signal removal (corresponding to experimental wash of cells to remove TNF), the adaptation coefficient k02_tnf_off=0.138 $\text{min}^{-}1$ (half-life of 5 min). We decided to take these values, because also in the file

Hoffmann_somrev.pdf the same half-life times of respectively 2.3 hrs and 5 min are given. Specifically, in the file Hoffmann_somrev.pdf, the authors wrote “We assume that following the signal onset there is a slow adaptation (k02_tnf_on = $1.2 \times 10^{-4} \times s^{-1}$; half-life of 2.3 hrs.), gradually reducing active IKK concentration. Signal removal (corresponding to experimental wash of cells to remove TNF) is modeled as adjustment of the adaptation coefficient k02_tnf_off to 0.18 s^{-1} (half-life of 5 min.)”. The value 0.18 s^{-1} implies half time is equal to 4 sec, which seem not physiological, and also makes that NF-κB may not respond to subsequent TNF pulses considered by Ashall et al.[2], because IκBα accumulates between pulses to a high level.

The model corresponding to knock-out of IκBβ and IκBε is obtained by setting tr2b= 0 and tr2e= 0.

1.1 Simulation protocol

The input into the signaling module is represented by the concentration of the active IKK. All simulations are started with IKK concentration equal to zero. Following ”equilibration” for 2400 min, IKK is raised as a step function to 0.1 μM. The initial concentration of the cytoplasmic NF-κB/IκBα complex is 0.1 μM and the initial concentrations of other variables are zero at the beginning of the equilibration phase.

Following the signal onset there is a slow adaptation k02_tnf_on , gradually reducing active IKK concentration. Signal removal (corresponding to experimental wash of cells to remove TNF) is modeled by adjustment of the adaptation coefficient to k02_tnf_off.

A summary of the considered stimulation protocols is given in S1 Table.

Table 3: **Parameter values for the Hoffmann et al. 2002 model.**

Parameter	Value	Units
a4	3.00E+01	μM^{-}1min^{-}1
d4	3.00E-02	min^{-}1
a5	3.00E+01	μM^{-}1min^{-}1
d5	3.00E-02	min^{-}1
a6	3.00E+01	μM^{-}1min^{-}1
d6	3.00E-02	min^{-}1
a1	1.35E+00	μM^{-}1min^{-}1
d1	7.50E-02	min^{-}1
r1	2.44E-01	min^{-}1
a2	3.60E-01	μM^{-}1min^{-}1
d2	1.05E-01	min^{-}1
r2	9.00E-02	min^{-}1
a3	5.40E-01	μM^{-}1min^{-}1
d3	1.05E-01	min^{-}1
r3	1.32E-01	min^{-}1
a7	1.11E+01	μM^{-}1min^{-}1
r4	1.22E+00	min^{-}1
a8	2.88E+00	μM^{-}1min^{-}1
r5	4.50E-01	min^{-}1
a9	4.20E+00	μM^{-}1min^{-}1
r6	6.60E-01	min^{-}1
tr2	9.90E-01	μM^{-}1min^{-}1
tr2a	9.24E-05	μM min^{-}1
tr2b	1.07E-05	μM min^{-}1
tr2e	7.62E-06	μM min^{-}1
tr3	1.68E-02	min^{-}1
tr1	2.45E-01	min^{-}1
deg1	6.78E-03	min^{-}1
deg4	1.35E-03	min^{-}1
tp1	1.80E-02	min^{-}1
tp2	1.20E-02	min^{-}1
k1	5.40E+00	min^{-}1
k01	4.80E-03	min^{-}1
k2	8.28E-01	min^{-}1
k02_tnf_on	4.98E-03	min^{-}1
k02_tnf_off	1.38E-01	min^{-}1
k_{cyt}	1	
k_{nuc}	1	

2 Ashall et al. 2009 model

The model contains 14 variables defined in Table 4.

Table 4: **Variables of the Ashall et al. 2009 model.**

Variable	Symbol	Description
$y(1)$	IKKn	neutral IKK
$y(2)$	IKKa	active IKK
$y(3)$	IKKi	inactive IKK
$y(4)$	NFκB	free cytoplasmic NF-κB
$y(5)$	nNFκB	free nuclear NF-κB
$y(6)$	IκBα	free cytoplasmic IκBα
$y(7)$	nIκBα	free nuclear IκBα
$y(8)$	A20	protein A20
$y(9)$	tIκBα	mRNA transcript IκBα
$y(10)$	tA20	mRNA transcript A20
$y(11)$	pIκBα	phosphorylated form of IκBα
$y(12)$	pIκBα|NFκB	phosphorylated form of IκBα and NF-κB complex
$y(13)$	IκBα|NFκB	cytoplasmic IκBα and NF-κB complex
$y(14)$	nIκBα|nNFκB	nuclear IκBα and NF-κB complex
	TNF	TNF stimulation level, 0 (TNF OFF) or 1 (TNF ON)

Differential equations for the Ashall et al. 2009 model are following

$$
\begin{aligned}
\text{IKKn} \quad y(1)' &= kp * \frac{kbA20}{kbA20 + TNF * y(8)} * y(3) - TNF * ka * y(1), && (1)\\
\text{IKKa} \quad y(2)' &= TNF * ka * y(1) - ki * y(2), && (2)\\
\text{IKKi} \quad y(3)' &= ki \times y(2) - kp * \frac{kbA20}{kbA20 + TNF * y(8)} * y(3), && (3)\\
\text{NF}\kappa\text{B} \quad y(4)' &= kd1a * y(13) - ka1a * y(6) * y(4) - ki1 * y(4) && (4)\\
&\quad + ke1 * y(5) + kt2a * y(12) + c5a * y(13), \\
\text{nNF}\kappa\text{B} \quad y(5)' &= kd1a * y(14) - ka1an * y(7) * y(5) + ki1 * kv * y(4) && (5)\\
&\quad - ke1 * kv * y(5), \\
\text{I}\kappa\text{B}\alpha \quad y(6)' &= kd1a * y(13) - ka1a * y(6) * y(4) + c2a * y(9) && (6)\\
&\quad - c4a * y(6) - ki3a * y(6) + ke3a * y(7) - kc1a * y(2) * y(6), \\
\text{nI}\kappa\text{B}\alpha \quad y(7)' &= kd1a * y(14) - ka1an * y(7) * y(5) - c4a * y(7) && (7)\\
&\quad + ki3a * kv * y(6) - ke3a * kv * y(7), \\
\text{A20} \quad y(8)' &= c2 * y(10) - c4 * y(8), && (8)\\
\text{tI}\kappa\text{B}\alpha \quad y(9)' &= c1a * \frac{y(5)^h}{y(5)^h + k^h} - c3a * y(9), && (9)\\
\text{tA20} \quad y(10)' &= c1 * \frac{y(5)^h}{y(5)^h + k^h} - c3 * y(10), && (10)\\
\text{pI}\kappa\text{B}\alpha \quad y(11)' &= kc1a * y(2) * y(6) - kt1a * y(11), && (11)\\
\text{pI}\kappa\text{B}\alpha|\text{NF}\kappa\text{B} \quad y(12)' &= kc2a * y(2) * y(13) - kt2a * y(12), && (12)\\
\text{I}\kappa\text{B}\alpha|\text{NF}\kappa\text{B} \quad y(13)' &= ka1a * y(6) * y(4) - kd1a * y(13) - c5a * y(13) && (13)\\
&\quad + ke2a * y(14) - kc2a * y(2) * y(13), \\
\text{nI}\kappa\text{B}\alpha|\text{nNF}\kappa\text{B} \quad y(14)' &= ka1an * y(7) * y(5) - kd1a * y(14) - ke2a * kv * y(14). && (14)
\end{aligned}
$$

The parameter values of the model used for numerical simulations are given in Table 5.

2.1 Simulation protocol

Total IKK (conserved by the model) is set equal to 0.08 μM and initialized in simulations by setting IKKn = 0.08 μM, whereas total NF-κB (also conserved by the model) is set equal to $NF = 0.08$ μM and initialized as cytoplasmic complex by setting IκBα|NF-κB =

0.08 μM. The levels of remaining species were initialised to zero. The presence or absence of TNF was accounted by setting TNF = 1 or TNF = 0, respectively. We analyze responses of the model to the six TNF stimulation protocols (the tonic stimulation and 5 pulsatile protocols) defined in S1 Table. The system was allowed to equilibrate for 4000-minutes in the absence of TNF before beginning of each of protocols. A20-deficient cells were modeled by setting the synthesis rate of A20 mRNA, c1, equal to zero.

Table 5: **Parameters of the Ashall et al. 2009 model.**

Parameter	Values	Unit
Base Module		
kv	3.3	
tv	2700	μm^3
ka1a	0.5	$\mu M^{-1}s^{-1}$
ka1an	0.5	$\mu M^{-1}s^{-1}$
kd1a	0.0005	s^{-1}
c1a	1.4×10^{-7}	$\mu M^{-1}s^{-1}$
c2a	0.5	s^{-1}
c3a	0.0003	s^{-1}
c4a	0.0005	s^{-1}
c5a	0.000022	s^{-1}
ki1	0.0026	s^{-1}
ke1	ki1/50	s^{-1}
ke2a	0.01	s^{-1}
ki3a	0.00067	s^{-1}
ke3a	ki3a/2	s^{-1}
Base Module IKK Interacting Parameters		
IKKfold	2	
kc1a	0.037*1*IKKfold	s^{-1}
kc2a	0.037*5*IKKfold	s^{-1}
kt1a	0.1	s^{-1}
kt2a	0.1	s^{-1}
IKK parameters		
ka	0.004	s^{-1}
ki	0.003	s^{-1}
kp	0.0006	s^{-1}
kbA20	0.0018	
A20 protein synthesis and degradation		
c1	1.4×10^{-7}	$\mu M^{-1}s^{-1}$
c2	0.5	s^{-1}
c3	0.00048	s^{-1}
c4	0.0045	s^{-1}
h	2	
k	0.065	

3 Murakawa et al. 2015 model

The model contains 7 variables and 14 parameters given in Table 6. The variables are cytoplasmic A20 y(1), A20 transcript y(2), active IKK y(3), cytoplasmic (IκBα|NFκB) complex y(4), free cytoplasmic IκBα y(5), IκBα transcript y(6) and free nuclear NF-κB y(7). The dynamics is described by the following system

$$\begin{aligned}
\text{A20} \quad y(1)' &= k_9 * y(2) - k_3 * y(1), & (1)\\
\text{A20 mRNA} \quad y(2)' &= k_{12} * y(7) - k_7 * y(2) * RC3H1A20, & (2)\\
\text{IKK} \quad y(3)' &= TNF * k_{10} * exp(-y(1)) + \frac{k_2}{k_2 + y(1)} - k_4 * y(3), & (3)\\
\text{I}\kappa\text{B}\alpha\text{-NF}\kappa\text{B} \quad y(4)' &= k_1 * y(7) * y(5) - k_{14} * y(4) - k_6 * y(3) * y(4), & (4)\\
\text{I}\kappa\text{B}\alpha \quad y(5)' &= k_{14} * y(4) - k_1 * y(7) * y(5) + k_{11} * y(6) - k_5 * y(5), & (5)\\
\text{I}\kappa\text{B}\alpha\text{ mRNA} \quad y(6)' &= k_{13} * y(7) - k_8 * y(6) * RC3H1I\kappa B\alpha, & (6)\\
\text{NF}\kappa\text{B} \quad y(7)' &= k_6 * y(3) * y(4) - k_1 * y(7) * y(5) + k_{14} * y(4). & (7)
\end{aligned}$$

The parameter values used for numerical simulations, taken from the Supplementary Material, Murakawa et al., 2015[3], are given in Table 6 and account for WT cells.

Table 6: **Parameters of the Murakawa et al. 2015 model and their numerical values (WT cells).**

Parameter	Values (au)	Description
k_1	9727.7	association rate of IκBα and NF-κB
k_2	0.0011844	basal activation rate of IKK
k_3	0.85927	degradation rate of A20
k_4	0.22329	inactivation rate of IKK
k_5	0.0055014	degradation rate of IκBα
k_6	0.0063041	IKK-dependent degradation rate of IκBα bound to NF-κB
k_7	0.00029974	degradation rate of A20 mRNA
k_8	0.00038679	degradation rate of IκBα mRNA
k_9	0.099907	synthesis rate of A20 protein
k_{10}	12.657	TNF-dependent activation rate of IKK
k_{11}	0.12952	synthesis rate of IκBα protein
k_{12}	0.098751	synthesis rate of A20 mRNA dependent on NF-κB
k_{13}	0.0015859	synthesis rate of IκBα mRNA dependent on NF-κB
k_{14}	39.284	dissociation rate of NF-κB|IκBα

3.1 Simulation protocol

The auxiliary parameters RC3H1A20 and RC3H1IκBα (present in the equations for y(2) and y(6)) were set equal to 1; in the original study these parameters were introduced to account for system perturbations. The input parameter TNF is either set 0 (absence of TNF) or 1 (presence of TNF). We analyze responses of the model to the six TNF stimulation protocols (the tonic stimulation and 5 pulsatile protocols) defined in S1 Table. Total NF-κB conserved by the model was set equal to 1.4454 (arbitrary units) and initialized as cytoplasmic complex by setting (IkBa|NFkB) = 1.4454, the remaining variables were set zero. The system was allowed to equilibrate for 1400 minutes in the absence of TNF before the beginning of each of the protocols. A20-deficient cells were modeled by setting the synthesis rate of A20 mRNA, k_{12}, equal to zero.

4 Krishna et al. 2006 model

The model is non-dimensional and contains 3 variables: free nuclear NF-κB, IκB mRNA and cytoplasmic protein IκB, denoted by $y(1)$, $y(2)$ and $y(3)$, respectively. The dynamics is governed by

$$\text{NF}\kappa\text{B} \qquad y(1)' = TR * A * \frac{1-y(1)}{\varepsilon + y(3)} - B * y(3)\frac{y(1)}{\delta + y(1)}, \tag{1}$$

$$\text{I}\kappa\text{B}\alpha \text{ mRNA} \qquad y(2)' = y(1)^2 - y(2), \tag{2}$$

$$\text{I}\kappa\text{B}\alpha \qquad y(3)' = y(2) - C(1-y(1))\frac{y(3)}{\varepsilon + y(3)}. \tag{3}$$

The original parameters values are $A = 0.007$, $B = 954.5$, $C = 0.035$, $\delta = 0.029$, and $\varepsilon = 2 \times 10^{-5}$.

4.1 Simulation protocol

The Krishna et al. 2006 model accounts only for IKK-driven IκBα degradation (originally parameter C governing IκBα degradation is assumed to be proportional to IKK considered as a stimulus); thus, in the absence of stimulation, IκBα may remain on an arbitrarily high level. As a consequence, the model may not reproduce responses to TNF pulses observed by Ashall et al.[2], just because at the second TNF pulse the level of IκBα is very high, and the system may not respond to subsequent TNF pulses. We found, however, that when parameter C is assumed constant, while parameter A, proportional to the source term for nuclear NF-κB, is considered as an input (TR=1 when TNF is on, and TR=0 for TNF off), the model possesses a natural (0,0,0) steady state. We analyze responses of the, so modified, model to the six TNF stimulation protocols (the tonic stimulation and 5 pulsatile protocols) defined in S1 Table.
