# Supplementary material for: A plausible identifiable model of the canonical NF-κB signaling pathway
Source: PLoS One. 2023 Jun 2;18(6):e0286416. doi: 10.1371/journal.pone.0286416 (PMC10237389; doi:10.1371/journal.pone.0286416)
Supplement: S2 Text — (PDF) [file pone.0286416.s014.pdf]

S2 Text

Model reduction, scaling and non-dimensionalization

featuring the article

A plausible identifiable model of the canonical NF-κB signaling pathway

by Joanna Jaruszewicz-Błońska, Ilona Kosiuk, Wiktor Prus, and Tomasz Lipniacki

all at the Institute of Fundamental Technological Research
of the Polish Academy of Sciences, Warsaw, Poland

Model reduction

We will reduce the original model of the NF-κB developed by Lipniacki et al.[1] consisting of 15 equations to a 6-variable model. Following the original notation, we use upper-case letters to denote molar concentrations of molecules and their complexes. Nuclear concentrations are denoted by subscript n; cytoplasmic concentrations are without any subscript. Concentration of the A20 and IκBα transcripts are denoted by subscript t.

Table A. List of the variables of the original model (1).

Variable	Definition
$IKKn$	cytoplasmic concentration of neutral form of IKK
$IKKa$	cytoplasmic concentration of active form of IKK
$IKKi$	cytoplasmic concentration of inactive form of IKK
$(IKKa\|I\kappa B\alpha)$	cytoplasmic complex of IKK active and IκBα
$(IKKa\|I\kappa B\alpha\|NF\kappa B)$	cytoplasmic complex of IKK active, IκBα and NF-κB
$NF\kappa B$	cytoplasmic NF-κB
$NF\kappa B_n$	nuclear NF-κB
$A20$	cytoplasmic A20 protein
$A20_t$	A20 transcript
$I\kappa B\alpha$	cytoplasmic IκBα
$I\kappa B\alpha_n$	nuclear IκBα
$I\kappa B\alpha_t$	IκBα transcript
$(I\kappa B\alpha\|NF\kappa B)$	cytoplasmic complex of IκBα and NF-κB
$(I\kappa B\alpha\|NF\kappa B)_n$	nuclear complex of IκBα and NF-κB
Cg	control early gene

The dynamics of the 15 variables listed in Table A is governed by the following system of equations

[1] Lipniacki T, Paszek P, Brasier AR, Luxon B, Kimmel M. Mathematical model of NF-κB regulatory module. J. Theor. Biol. 2004; 228(2): 195–215. doi: 10.1016/j.jtbi.2004.01.001. PMID: 15094015.

$$
\begin{aligned}
IKKn' &= k_{prod} - (k_{deg} + T_R k_1) IKKn, && (1.1)\\
IKKa' &= T_R k_1 IKKn - k_3 IKK_a - T_R k_2 IKKa \cdot A20 && (1.2)\\
&\quad - k_{deg} IKKa - a_2 IKKa \cdot I\kappa B\alpha + t_1 (IKKa|I\kappa B\alpha)\\
&\quad - a_3 IKKa \cdot (I\kappa B\alpha|NF\kappa B) + t_2 (IKKa|I\kappa B\alpha|NF\kappa B)\\
IKKi' &= k_3 IKKa + T_R k_2 IKKa \cdot A20 - k_{deg} IKKi && (1.3)\\
(IKKa|I\kappa B\alpha)' &= a_2 IKKa \cdot I\kappa B\alpha - t_1 (IKKa|I\kappa B\alpha) && (1.4)\\
(IKKa|I\kappa B\alpha|NF\kappa B)' &= a_3 IKKa \cdot (I\kappa B\alpha|NF\kappa B) && (1.5)\\
&\quad - t_2 (IKKa|I\kappa B\alpha|NF\kappa B)\\
NF\kappa B' &= c_{6a} (I\kappa B\alpha|NF\kappa B) - a_1 NF\kappa B \cdot I\kappa B\alpha && (1.6)\\
&\quad + t_2 (IKKa|I\kappa B\alpha|NF\kappa B) - i_1 NF\kappa B,\\
NF\kappa B_n' &= i_1 k_v NF\kappa B - a_1 NF\kappa B_n \cdot I\kappa B\alpha_n && (1.7)\\
A20' &= c_4 A20_t - c_5 A20 && (1.8)\\
A20_t' &= c_2 + c_1 NF\kappa B_n - c_3 A20_t && (1.9)\\
I\kappa B\alpha' &= -a_2 IKKa \cdot I\kappa B\alpha - a_1 NF\kappa B \cdot I\kappa B\alpha && (1.10)\\
&\quad + c_{4a} I\kappa B\alpha_t - c_{5a} I\kappa B\alpha - i_{1a} I\kappa B\alpha + e_{1a} I\kappa B\alpha_n\\
I\kappa B\alpha_n' &= -a_1 NF\kappa B_n \cdot I\kappa B\alpha_n + i_{1a} k_v I\kappa B\alpha - e_{1a} k_v I\kappa B\alpha_n && (1.11)\\
I\kappa B\alpha_t' &= c_{1a} NF\kappa B_n - c_{3a} I\kappa B\alpha_t && (1.12)\\
(I\kappa B\alpha|NF\kappa B)' &= a_1 NF\kappa B \cdot I\kappa B\alpha - c_{6a} (I\kappa B\alpha|NF\kappa B) && (1.13)\\
&\quad - a_3 IKKa \cdot (I\kappa B\alpha|NF\kappa B) + e_{2a} (I\kappa B\alpha|NF\kappa B)_n\\
(I\kappa B\alpha|NF\kappa B)_n' &= a_1 NF\kappa B_n \cdot I\kappa B\alpha_n - e_{2a} k_v (I\kappa B\alpha|NF\kappa B)_n && (1.14)\\
Cg' &= c_{2c} + c_{1c} NF\kappa B_n - c_{3c} Cg. && (1.15)
\end{aligned}
$$

The original parameter values are given in Table B.

Table B. Parameters of the original model (1) and their default numerical values.

Parameter	Value	Unit	Description
C_T	0.3	μM	total NF-κB concentration w/r/t nuclear volume
k_v	5		ratio of cytoplasmic to nuclear volume
c_1	0.0000005	s^{-1}	inducible A20 mRNA synthesis
c_2	0.0	μMs^{-1}	constitutive A20 mRNA synthesis
c_3	0.0004	s^{-1}	A20 mRNA degradation
c_4	0.5	s^{-1}	A20 translation
c_5	0.0003	s^{-1}	A20 degradation
k_1	0.0025	s^{-1}	IKK activation caused by TNF
k_2	0.1	s^{-1}	IKK inactivation caused by $A20$
k_3	0.0015	s^{-1}	IKK spontaneous inactivation
k_{prod}	0.000025	μMs^{-1}	IKKn production rate
k_{deg}	0.000125	s^{-1}	degradation of IKKa, IKKn and IKKi
a_2	0.2	μM$^{-1}s^{-1}$	IKK IκBα association
a_1	0.5	μM$^{-1}s^{-1}$	IκBα NF-κB association
a_3	1	μM$^{-1}s^{-1}$	IKK (IκBα | NF-κB) association
t_1	0.1	s^{-1}	degradation of IκBα in (IKK | IκBα) complex
t_2	0.1	s^{-1}	degradation of IκBα in (IKK | IκBα | NF-κB) complex
c_{1a}	0.0000005	s^{-1}	inducible IκBα mRNA synthesis
c_{2a}	0.0	μMs^{-1}	constitutive mRNA IκBα synthesis
c_{3a}	0.0004	s^{-1}	IκBα mRNA degradation
c_{4a}	0.5	s^{-1}	IκBα translation
c_{5a}	0.0001	s^{-1}	IκBα degradation
c_{6a}	0.00002	s^{-1}	degradation IκBα in (IκBα | NF-κB) complex
i_1	0.0025	s^{-1}	NF-κB nuclear import
e_{2a}	0.01	s^{-1}	(IκBα | NF-κB) nuclear export
i_{1a}	0.001	s^{-1}	IκBα nuclear import
e_{1a}	0.0005	s^{-1}	IκBα nuclear export
c_{1c}	0.0000005	s^{-1}	inducible transcription
c_{2c}	0.	μMs^{-1}	constitutive transcription
c_{3c}	0.0004	s^{-1}	control gene mRNA degradation

The model reduction is carried out in six steps discussed below.

Step 1. Elimination of equations for Cg and $IKKi$. Elimination of terms with zero rates.

We eliminate Cg and $IKKi$ as non-essential readouts not influencing the dynamics of the remaining 13 variables. We eliminate terms having in the original model (1) zero rates, i.e., c_2, c_{2a}, c_{2c}. In addition, we set $c_{6a} = 0$.

Step 2. Elimination of equations (1.4) and (1.5) describing the kinetics of $(IKKa|I\kappa B\alpha)$ and $(IKKa|I\kappa B\alpha|NF\kappa B)$ complexes.

In this step, as well as in steps 3 and 4, we eliminate fast variables, which describe either short lasting complexes or proteins that either rapidly bind to other proteins or are rapidly translocated to the other compartment.

Binding of IKKa to $I\kappa B\alpha$ and $(I\kappa B\alpha|NF\kappa B)$ results in $I\kappa B\alpha$ degradation. Since $t_1 = t_2 = 0.1\ s^{-1}$ complexes $(IKKa|I\kappa B\alpha)$ and $(IKKa|I\kappa B\alpha|NF\kappa B)$ are short lasting, with repect to the characteristic scale of regulatory process. Thus, one can neglect these intermediate steps in $I\kappa B\alpha$ degradation and assume that IKKa catalyzes $I\kappa B\alpha$ degradation with rates a_2 and a_3 equal to the rates of complex formation, which leads to the following 11-dimensional system

$$IKKn' = k_{prod} - (k_{deg} + T_R k_1) IKKn, \tag{2.1}$$

$$IKKa' = T_R k_1 IKKn - k_3 IKKa - T_R k_2 IKKa \cdot A20 - k_{deg} IKKa, \tag{2.2}$$

$$NF\kappa B' = a_3 IKKa \cdot (I\kappa B\alpha|NF\kappa B) - a_1 NF\kappa B \cdot I\kappa B\alpha - i_1 NF\kappa B, \tag{2.3}$$

$$NF\kappa B_n' = i_1 k_v NF\kappa B - a_1 NF\kappa B_n \cdot I\kappa B\alpha_n, \tag{2.4}$$

$$A20' = c_4 A20_t - c_5 A20, \tag{2.5}$$

$$A20_t' = c_1 NF\kappa B_n - c_3 A20_t, \tag{2.6}$$

$$\begin{aligned} I\kappa B\alpha' = & -a_2 IKKa \cdot I\kappa B\alpha - a_1 NF\kappa B \cdot I\kappa B\alpha + c_{4a} I\kappa B\alpha_t \\ & - c_{5a} I\kappa B\alpha - i_{1a} I\kappa B\alpha + e_{1a} I\kappa B\alpha_n, \end{aligned} \tag{2.7}$$

$$I\kappa B\alpha_n' = i_{1a} k_v I\kappa B\alpha - a_1 NF\kappa B_n \cdot I\kappa B\alpha_n - e_{1a} k_v I\kappa B\alpha_n, \tag{2.8}$$

$$I\kappa B\alpha_t' = c_{1a} NF\kappa B_n - c_{3a} I\kappa B\alpha_t, \tag{2.9}$$

$$\begin{aligned} (I\kappa B\alpha|NF\kappa B)' = & a_1 NF\kappa B \cdot I\kappa B\alpha - a_3 IKKa \cdot (I\kappa B\alpha|NF\kappa B) \\ & + e_{2a} (I\kappa B\alpha|NF\kappa B)_n, \end{aligned} \tag{2.10}$$

$$(I\kappa B\alpha|NF\kappa B)_n' = a_1 NF\kappa B_n \cdot I\kappa B\alpha_n - e_{2a} k_v (I\kappa B\alpha|NF\kappa B)_n. \tag{2.11}$$

Step 3. Elimination of the equations for cytoplasmic $NF\kappa B$ and nuclear $I\kappa B\alpha_n$

In the equation (2.3) for the cytoplasmic $NF\kappa B$ the first term represents liberation NF-κB due to degradation of IκBα in the cytoplasmic $(I\kappa B\alpha|NF\kappa B)$ complexes, the second term represents $NF\kappa B$ depletion due to formation of complexes with IκBα. The last term describes transport of free cytoplasmic NF-κB to the nucleus. As these two last processes are fast we may in approximation neglect $NF\kappa B'$. We assume the steady state of free cytoplasmic NF-κB is reached ($NF\kappa B' = 0$) and thus obtain

$$NF\kappa B = \frac{a_3 IKKa \cdot (I\kappa B\alpha|NF\kappa B)}{a_1 I\kappa B\alpha + i_1}. \tag{3}$$

In the equation (2.8) for the nuclear free $I\kappa B\alpha_n$ the first term describes IκBα transport to the nucleus, the second term describes depletion of $I\kappa B\alpha_n$ due to formation of the nuclear complexes $(I\kappa B\alpha|NF\kappa B)_n$, while the last term represent the IκBα transport out of the nucleus. Similarly, because these two last processes are fast, we will in approximation neglect $I\kappa B\alpha_n'$ and thus obtain

$$I\kappa B\alpha_n = \frac{i_{1a}k_v I\kappa B\alpha}{a_1 NF\kappa B_n + e_{1a}k_v}. \tag{4}$$

Consequently, using (3) and (4), we obtain the following system of 9 equations

$$IKKn' = k_{prod} - (k_{deg} + T_R k_1)IKKn, \tag{5.1}$$

$$IKKa' = T_R k_1 IKKn - k_3 IKKa - T_R k_2 IKKa \cdot A20 - k_{deg} IKKa, \tag{5.2}$$

$$NF\kappa B_n' = k_v a_3 IKKa \cdot (I\kappa B\alpha|NF\kappa B)\frac{\mu}{I\kappa B\alpha + \mu} - i_{1a}k_v I\kappa B\alpha \frac{NF\kappa B_n}{NF\kappa B_n + \nu}, \tag{5.3}$$

$$A20' = c_4 A20_t - c_5 A20, \tag{5.4}$$

$$A20_t' = c_1 NF\kappa B_n - c_3 A20_t, \tag{5.5}$$

$$\begin{aligned} I\kappa B\alpha' = {} & c_{4a} I\kappa B\alpha_t - a_2 IKKa \cdot I\kappa B\alpha - c_{5a} I\kappa B\alpha \\ & - a_3 IKKa \cdot (I\kappa B\alpha|NF\kappa B)\frac{I\kappa B\alpha}{I\kappa B\alpha + \mu} - i_{1a} I\kappa B\alpha \frac{NF\kappa B_n}{NF\kappa B_n + \nu}, \end{aligned} \tag{5.6}$$

$$I\kappa B\alpha_t' = c_{1a} NF\kappa B_n - c_{3a} I\kappa B\alpha_t, \tag{5.7}$$

$$(I\kappa B\alpha|NF\kappa B)' = - a_3 IKKa \cdot (I\kappa B\alpha|NF\kappa B)\frac{\mu}{I\kappa B\alpha + \mu} + e_{2a}(I\kappa B\alpha|NF\kappa B)_n, \tag{5.8}$$

$$(I\kappa B\alpha|NF\kappa B)_n' = i_{1a}k_v I\kappa B\alpha \frac{NF\kappa B_n}{NF\kappa B_n + \nu} - e_{2a}k_v (I\kappa B\alpha|NF\kappa B)_n, \tag{5.9}$$

where $\mu := \frac{i_1}{a_1}$ and $\nu := \frac{e_{1a}k_v}{a_1}$.

Step 4. Elimination of equation (5.9) for the nuclear complex $(I\kappa B\alpha|NF\kappa B)_n$

The equation (5.9) contains two terms: the first one describes formation of $(I\kappa B\alpha|NF\kappa B)_n$ nuclear complexes and the second one describes their transport out of the nucleus. Since these processes are relatively fast in approximation we neglect $(I\kappa B\alpha|NF\kappa B)_n'$, and thus obtain $e_{2a}k_v(I\kappa B\alpha|NF\kappa B)_n = i_{1a}k_v I\kappa B\alpha \frac{NF\kappa B_n}{NF\kappa B_n + \nu}$. This allows us to eliminate equation (5.9) and replace equation (5.8) by

$$(I\kappa B\alpha|NF\kappa B)' \;=\; -a_3 IKKa \cdot (I\kappa B\alpha|NF\kappa B)\frac{\mu}{I\kappa B\alpha + \mu} + i_{1a} I\kappa B\alpha \frac{NF\kappa B_n}{NF\kappa B_n + \nu}. \tag{6}$$

Step 5. Elimination of cytoplasmic complex $(I\kappa B\alpha|NF\kappa B)$.

The resulting 8-variable system (5.1)–(5.7) and (6) has one conservation law: $NF\kappa B_n' + k_v(I\kappa B\alpha|NF\kappa B)' = 0$. Hence, we obtain $(I\kappa B\alpha|NF\kappa B) = (C_T - NF\kappa B_n)/k_v$ with the maximal concentration of nuclear NF-κB denoted by C_T=0.3μM. This in turn corresponds to the cytoplasmic concentration of 0.06μM, as it is assumed that the cytoplasmic volume is $k_v = 5$ times larger. Note that two proteins IκBα and NF-κB may form complexes in the cytoplasm, and although the variable describing NF-κB | IκBα complexes will not explicitly be present in the reduced model, the concentration of these complexes is given by $(C_T - NF\kappa B_n)/k_v$.

The resulting reduced 7-variable system becomes

$$IKKn' = k_{prod} - (k_{deg} + T_R k_1) IKKn, \tag{7.1}$$

$$IKKa' = T_R k_1 IKKn - k_3 IKKa - T_R k_2 IKKa \cdot A20 - k_{deg} IKKa, \tag{7.2}$$

$$NF\kappa B_n' = a_3 IKKa \cdot (C_T - NF\kappa B_n) \frac{\mu}{I\kappa B\alpha + \mu} - i_{1a} k_v I\kappa B\alpha \frac{NF\kappa B_n}{NF\kappa B_n + \nu}, \tag{7.3}$$

$$A20' = c_4 A20_t - c_5 A20, \tag{7.4}$$

$$A20_t' = c_1 NF\kappa B_n - c_3 A20_t, \tag{7.5}$$

$$\begin{aligned} I\kappa B\alpha' = {} & c_{4a} I\kappa B\alpha_t - a_2 IKKa \cdot I\kappa B\alpha - c_{5a} I\kappa B\alpha \\ & - \frac{a_3}{k_v} IKKa (C_T - NF\kappa B_n) \frac{I\kappa B\alpha}{I\kappa B\alpha + \mu} - i_{1a} I\kappa B\alpha \frac{NF\kappa B_n}{NF\kappa B_n + \nu}, \end{aligned} \tag{7.6}$$

$$I\kappa B\alpha_t' = c_{1a} NF\kappa B_n - c_{3a} I\kappa B\alpha_t. \tag{7.7}$$

Step 6. Elimination of $A20_t$

In approximation we replace the equations (7.4)–(7.5) with a single new equation for protein A20

$$A20' = c_{prod} NF\kappa B_n - c_{deg} A20, \tag{8}$$

where $c_{prod} := \frac{c_1 c_4}{(c_3 + c_5)}$ and $c_{deg} := \frac{c_3 c_5}{(c_3 + c_5)}$ are effective coefficients A20 protein synthesis and A20 protein coefficient, respectively. These coefficients assure that for constant $NF\kappa B_n$ the equilibrium A20 protein concentration is

$$A20_{eq} = \frac{c_{prod} NF\kappa B_n}{c_{deg}} = \frac{c_1 c_4 NF\kappa B_n}{c_3 c_5} \tag{9}$$

equal to the equilibrium concentration following from the equations (7.4)–(7.5). Additionally, according to the equations (7.4)–(7.5) the characteristic time of reaching equilibrium concentration for A20 mRNA is $1/c_3$, while the characteristic time in which A20 protein reaches equilibrium concentration is $1/c_5$. A sum of these times is $\frac{c_3 + c_5}{c_3 c_5} = 1/c_{deg}$.

The resulting final reduced system is 6-dimensional and its dynamics is governed by

$$
\begin{aligned}
IKKn' &= k_{prod} - (k_{deg} + T_R k_1) IKKn, \\
IKKa' &= T_R k_1 IKKn - k_3 IKKa - T_R k_2 IKKa \cdot A20 - k_{deg} IKKa, \\
NF\kappa B_n' &= a_3 IKKa \cdot (C_T - NF\kappa B_n) \frac{\mu}{I\kappa B\alpha + \mu} - i_{1a} k_v I\kappa B\alpha \frac{NF\kappa B_n}{NF\kappa B_n + \nu}, \\
A20' &= c_{prod} NF\kappa B_n - c_{deg} A20, \qquad (10) \\
I\kappa B\alpha' &= c_{4a} I\kappa B\alpha_t - a_2 IKKa \cdot I\kappa B\alpha - \frac{a_3}{k_v} IKKa (C_T - NF\kappa B_n) \frac{I\kappa B\alpha}{I\kappa B\alpha + \mu} \\
&\quad - c_{5a} I\kappa B\alpha - i_{1a} I\kappa B\alpha \frac{NF\kappa B_n}{NF\kappa B_n + \nu} \\
I\kappa B\alpha_t' &= c_{1a} NF\kappa B_n - c_{3a} I\kappa B\alpha_t.
\end{aligned}
$$

The parameters of the reduced model (10) and their numerical values obtained from the model reduction are given in Table D.

Table C. Variables of the reduced model (10).

Variable	Definition
$IKKn$	cytoplasmic concentration of neutral form of IKK
$IKKa$	cytoplasmic concentration of active form of IKK
$NF\kappa B_n$	free nuclear NF-κB
$A20$	A20 protein
$I\kappa B\alpha$	free cytoplasmic IκBα protein
$I\kappa B\alpha_t$	IκBα transcript

Table D. Parameters of the reduced model (10) and their numerical values obtained from the model reduction.

Parameter	Value	Unit	Description
C_T	0.3	μM	total NF-κB concentration w/r/t nuclear volume
k_v	5		ratio of cytoplasmic to nuclear volume
k_1	0.0025	s^{-1}	IKK activation caused by TNF
k_2	0.1	s^{-1}	IKK inactivation caused by A20
k_3	0.0015	s^{-1}	IKK spontanouous inactivation
k_{prod}	0.000025	μMs^{-1}	IKKn production rate
k_{deg}	0.000125	s^{-1}	degradation of IKKa and IKKn
μ	0.005	μM	Michaelis constant for inhibition of nuclear NF-κB by IκBα
ν	0.005	μM	Michaelis constant for the export of NF-κB from the nucleus
c_{prod}	0.000357	s^{-1}	effective A20 protein synthesis coefficient
c_{deg}	0.000171	s^{-1}	effective A20 degradation coefficient
a_2	0.2	μM$^{-1}s^{-1}$	IKK IκBα association leading to IκBα degradation
a_3	1	μM$^{-1}s^{-1}$	IKK (IκBα | NF-κB) association leading to IκBα degradation
c_{1a}	0.0000005	s^{-1}	inducible IκBα mRNA synthesis
c_{3a}	0.0004	s^{-1}	IκBα mRNA degradation
c_{4a}	0.5	s^{-1}	IκBα translation
c_{5a}	0.0001	s^{-1}	IκBα degradation
i_{1a}	0.001	s^{-1}	IκBα nuclear import

Model scaling and non-dimensionalization

The available data in most cases lacks the absolute quantification. Therefore, the dependent variables must be non-dimensionalized in order to make the model identifiable. We introduce the following scaling leaving time the only dimensional variable.

As a first step, we scale

$$NF\kappa B_n := k_v N\tilde{F}\kappa B, \tag{11}$$

which results in

$$\begin{aligned}
IKKn' &= k_{prod} - (k_{deg} + T_R k_1) IKKn, \\
IKKa' &= T_R k_1 IKKn - k_3 IKKa - T_R k_2 IKKa \cdot A20 - k_{deg} IKKa, \\
k_v N\tilde{F}\kappa B_n' &= a_3 IKKa \cdot (C_T - k_v N\tilde{F}\kappa B_n) \frac{\mu}{I\kappa B\alpha + \mu} - i_{1a} k_v I\kappa B\alpha \frac{k_v N\tilde{F}\kappa B_n}{k_v N\tilde{F}\kappa B_n + \nu}, \\
A20' &= c_{prod} k_v N\tilde{F}\kappa B_n - c_{deg} A20, \\
I\kappa B\alpha' &= c_{4a} I\kappa B\alpha_t - a_2 IKKa \cdot I\kappa B\alpha - \frac{a_3}{k_v} IKKa (C_T - k_v N\tilde{F}\kappa B_n) \frac{I\kappa B\alpha}{I\kappa B\alpha + \mu} \\
&\quad - c_{5a} I\kappa B\alpha - i_{1a} I\kappa B\alpha \frac{k_v N\tilde{F}\kappa B_n}{k_v N\tilde{F}\kappa B_n + \nu}, \\
I\kappa B\alpha_t' &= c_{1a} k_v N\tilde{F}\kappa B_n - c_{3a} I\kappa B\alpha_t.
\end{aligned} \tag{12}$$

We re-write the system (12) as

$$\begin{aligned}
IKKn' &= k_{prod} - (k_{deg} + T_R k_1) IKKn, \\
IKKa' &= T_R k_1 IKKn - k_3 IKKa - T_R k_2 IKKa \cdot A20 - k_{deg} IKKa, \\
NF\kappa B_n' &= a_3 IKKa \cdot (C_0 - NF\kappa B_n) \frac{\mu}{I\kappa B\alpha + \mu} - i_{1a} I\kappa B\alpha \frac{NF\kappa B_n}{NF\kappa B_n + \epsilon}, \\
A20' &= C_{prod} NF\kappa B_n - c_{deg} A20, \\
I\kappa B\alpha' &= c_{4a} I\kappa B\alpha_t - a_2 IKKa \cdot I\kappa B\alpha - a_3 IKKa (C_0 - NF\kappa B_n) \frac{I\kappa B\alpha}{I\kappa B\alpha + \mu} \\
&\quad - c_{5a} I\kappa B\alpha - i_{1a} I\kappa B\alpha \frac{NF\kappa B_n}{NF\kappa B_n + \epsilon}, \\
I\kappa B\alpha_t' &= C_{1a} NF\kappa B_n - c_{3a} I\kappa B\alpha_t,
\end{aligned} \tag{13}$$

where

$$C_0 := C_T/k_v,\ C_{prod} := c_{prod} k_v,\ C_{1a} := c_{1a} k_v,\ \epsilon = \nu/k_v$$

and $\tilde{}$ has been dropped.

Next we introduce the following scaling

$$\begin{aligned}
IKKn = \tfrac{k_{prod}}{k_{deg}} I\tilde{K}Kn, \quad IKKa = \tfrac{k_{prod}}{k_{deg}} I\tilde{K}Ka, \quad NF\kappa B_n = C_0 N\tilde{F}\kappa B_n, \\
A20 = \tfrac{C_{prod} C_0}{c_{deg}} A\tilde{2}0, \qquad I\kappa B\alpha = C_0 I\kappa \tilde{B}\alpha, \qquad I\kappa B\alpha_t = \tfrac{C_{1a} C_0}{c_{3a}} I\kappa \tilde{B}\alpha_t,
\end{aligned} \tag{14}$$

where $\tilde{}$ denotes the non-dimensional variable, and will be omitted from here on. Inserting

(14) into system (13) and dropping ˜, we obtain

$$
\begin{aligned}
IKKn' &= k_{deg}(1-IKKn)-T_R k_1 IKKn,\\
IKKa' &= T_R k_1 IKKn-(k_3+k_{deg}+T_R\bar{k}_2 A20)IKKa,\\
NF\kappa B_n' &= \bar{a}_3 IKKa\cdot(1-NF\kappa B_n)\frac{\delta}{I\kappa B\alpha+\delta}-i_{1a}I\kappa B\alpha\frac{NF\kappa B_n}{NF\kappa B_n+\varepsilon},\\
A20' &= c_{deg}(NF\kappa B_n-A20),\\
I\kappa B\alpha' &= \bar{c}_{4a}I\kappa B\alpha_t-\bar{a}_2 IKKa\cdot I\kappa B\alpha-\bar{a}_3 IKKa(1-NF\kappa B_n)\frac{I\kappa B\alpha}{I\kappa B\alpha+\delta}\\
&\quad -c_{5a}I\kappa B\alpha-i_{1a}I\kappa B\alpha\frac{NF\kappa B_n}{NF\kappa B_n+\varepsilon},\\
I\kappa B\alpha_t' &= c_{3a}(NF\kappa B_n-I\kappa B\alpha_t),
\end{aligned}
\tag{15}
$$

where

$$
\begin{aligned}
\bar{k}_2 &:= k_2\tfrac{c_{prod}}{c_{deg}}C_T, \qquad \delta := \tfrac{\mu}{C_0}, \qquad \varepsilon := \tfrac{\epsilon}{C_0}=\tfrac{\nu}{C_T},\\
\bar{a}_2 &:= a_2\tfrac{k_{prod}}{k_{deg}}, \qquad \bar{a}_3 := a_3\tfrac{k_{prod}}{k_{deg}}, \qquad \bar{c}_{4a} := \tfrac{c_{4a}}{c_{3a}}C_{1a}.
\end{aligned}
\tag{16}
$$

In the consequence of nondimensionalization the number of parameters has been reduced from 18 to 13, see Table E. For convenience , we will drop ¯ in the notation of the parameters defined in (16), but it is to be understood that the parameters k_2, a_2, a_3, c_{4a} of the reduced rescaled model are appropriately scaled versions of the corresponding parameters of the original model.

Table E. Parameters of the rescaled reduced model (15) and their numerical values.

Parameter	Value	Unit	Description
k_{deg}	0.000125	s^{-1}	degradation of IKKn and IKKa
k_1	0.0025	s^{-1}	activation of IKKn caused by TNF
k_3	0.0015	s^{-1}	spontaneous inactivation of IKKa
k_2	0.0625	s^{-1}	inactivation of IKKa induced by A20
a_3	0.2	s^{-1}	IKK (IκBα | NF-κB) association leading to IκBα degradation
δ	0.0833		Michaelis type constant
ε	0.01667		Michaelis type constant
c_{deg}	0.000171	s^{-1}	effective A20 degradation
c_{4a}	0.0031	s^{-1}	IκBα translation
a_2	0.04	s^{-1}	IκBα degradation induced by IKKa
c_{5a}	0.0001	s^{-1}	IκBα degradation
i_{1a}	0.001	s^{-1}	IκBα nuclear import leading to NF-κB removal from the nucleus
c_{3a}	0.0004	s^{-1}	IκBα transcription and mRNA degradation
