# Supplementary material for: A plausible identifiable model of the canonical NF-κB signaling pathway
Source: PLoS One. 2023 Jun 2;18(6):e0286416. doi: 10.1371/journal.pone.0286416 (PMC10237389; doi:10.1371/journal.pone.0286416)
Supplement: Attachment — Submitted filename: Jaruszewicz_et_al__Resubmission__Response_to_Reviews.pdf [file pone.0286416.s016.pdf]

Tomasz Lipniacki
Department of Biosystems and Soft Matter
Institute of Fundamental Technological Research
Polish Academy of Sciences

Pawińskiego 5B, 02-106 Warsaw, Poland
Phone: +48 22 8261280
E-mail: tlipnia@ippt.pan.pl
Website: https://pmbm.ippt.pan.pl

Subject: **Resubmission of the revised manuscript PCOMPBIOL-D-22-00862** Jan 16, 2023

Dear Editor, Dear Reviewers,

Thank you for your thorough reading and comments on our manuscript "A plausible identifiable model of the canonical NF-κB signaling pathway". In this document, we provide a concise description of the amendments (directly below) and detailed point-by-point responses to your comments (on following pages).

The main amendments and additions are the following:

1. Based on 6 considered TNF stimulation protocols (taken from the literature), we discuss the ability of both the original and the reduced model to reproduce experimental results. In response to Editor's comments below, we included a figure with comparison of model trajectories for the Reviewers' and the Editor's convenience.

2. In Supplementary Figure S1 we compare responses of the Hoffmann *et al.* 2002 model and the Lipniacki *et al.* 2004 model to all considered protocols. The figure shows qualitative agreement (for WT cells) between these two experimentally constrained models (the earlier model, however, is not able to respond to 3 TNF pulses separated by 200-min long intervals). The important differences between these two models are as follows:

   - The Hoffmann *et al.* 2002 model accounts for 3 IκB isoforms, whereas the Lipniacki *et al.* 2004 model replaces them by a single dominant IκB isoform, IκBα.
   - In contrast to the Hoffmann *et al.* 2002 model, the Lipniacki *et al.* 2004 model accounts for A20, an NF-κB-inducible inhibitor of NF-κB. A20 attenuates activity of IKK, mediating another crucial negative feedback loop. As a consequence, the latter model can reproduce qualitatively different trajectories of WT and A20-deficient cells in response to tonic TNF stimulation.

3. In Supplementary Figure S6 we analyze the ability of the Krishna *et al.* 2006 model to reproduce trajectories of the Lipniacki *et al.* 2004 model (the original model of our study). The figure shows that the Krishna *et al.* 2006 model cannot be fully fitted to trajectories obtained with the Lipniacki *et al.* 2004 model. The NF-κB trajectory is satisfactorily reproduced, whereas the IκBα trajectory is not. The Krishna *et al.* 3-variable model was the basis for the Jensen & Krishna 2012 model. This helped us to discuss the "advance over previous reduced models" mentioned by Referee 3, as requested by the Editor.

A detailed point-by-point response to the Editor's and Reviewers' comments is provided below. All changes in the manuscript are highlighted in color in another separate PDF file.

We hope that you will find the revised manuscript suitable for publication in *PLOS Computational Biology.*

Sincerely,

Tomasz Lipniacki
Professor and Head

Response to the Editor's comments

> As with all papers reviewed by the journal, your manuscript was reviewed by members of the editorial board and by several independent reviewers. In light of the reviews (below this email), we would like to invite the resubmission of a significantly-revised version that takes into account the reviewers' comments. In particular, all three reviewers have expressed doubts about the significance and novelty of the presented work and model reduction. **It is important to address how the present model represents and advance over previous reduced models that have been presented in the literature, for example, those mentioned by reviewer 3**. Reviewer 1 also raises the important point that the **model should be demonstrated to perform well with experiment data**, not just synthetic data derived from other models.

Response: Advance over previous reduced models. In the Discussion section of the revised manuscript we compare our reduced model with the existing reduced models. Below we include a more detailed discussion, being aware of the fact that the previous models were proposed at the time, when much less experimental data were available.

In two out of three of papers mentioned by Referee 3, the reduced models are defined explicitly.

(A) The first one is the Krishna *et al.* 2006 model (the first reduced NF-ϰB model, based on Hoffmann et al. 2002 model) that consists of 3 non-dimensional equations for free nuclear NF-ϰB (N_{n}), IϰBα transcript (I_{m}), and free cytoplasmic IϰBα (I):

$$
\begin{aligned}
\dot{N}_{\mathrm{n}} &= A\tfrac{(1-N_{\mathrm{n}})}{\varepsilon+I} - BI\tfrac{N_{\mathrm{n}}}{\delta+N_{\mathrm{n}}},\\
\dot{I}_{\mathrm{m}} &= N_{\mathrm{n}}^2 - I_{\mathrm{m}},\\
\dot{I} &= I_{\mathrm{m}} - C(1-N_{\mathrm{n}})\tfrac{I}{\varepsilon+I}.
\end{aligned}
$$

The model has only 5 parameters, and is thus possibly identifiable based on experimental trajectories of WT cells, but the authors are not discussing this. The key difference between our reduced model and the model by Krishna *et al.* is that the Krishna *et al.* 2006 model has no variable for A20 protein, and thus NF-ϰB dynamics is controlled solely by IϰBα. It is known, however, that an A20-mediated negative loop is key to oscillatory NF-ϰB responses — in A20 KO cells, IKK activity is elevated, causing that IϰBα may not be replenished, and, as a consequence, NF-ϰB remains in the nucleus for hours (see Lee *et al.* 2000,

DOI: 10.1126/science.289.5488.2350, Fig. 3). A20 is also inhibitor of kinase TBK1, which activates IRF3, the other critical transcription factor regulating innate immune responses. As a consequence A20 regulates the crosstalk between NF-κB and IRF3, that are both activated upon recognition of pathogens, and are both needed for synthesis of interferons, produced to warn surounding cells about infection. The fact that Krishna *et al.* 2006 model exhibits well-pronounced oscillations implies that A20 is in a sense present implicitly (which is OK), but obviously such a model may not reproduce behavior of cells with A20 knockout without some additional assumptions.

The Krishna *et al.* 2006 model was motivated by the study of Nelson *et al.* 2004 (DOI: 10.1126/science.1099962), in which the single-cell responses of SK-N-AS cells expressing a fluorescently tagged IκBα in addition to fluorescently tagged NF-κB component, RelA-DsRed. In particular, Krishna *et al.* compares their model trajectories from Fig. 2 (see below) with trajectories shown in Nelson *et al.* in Fig. 3B (left subpanel).

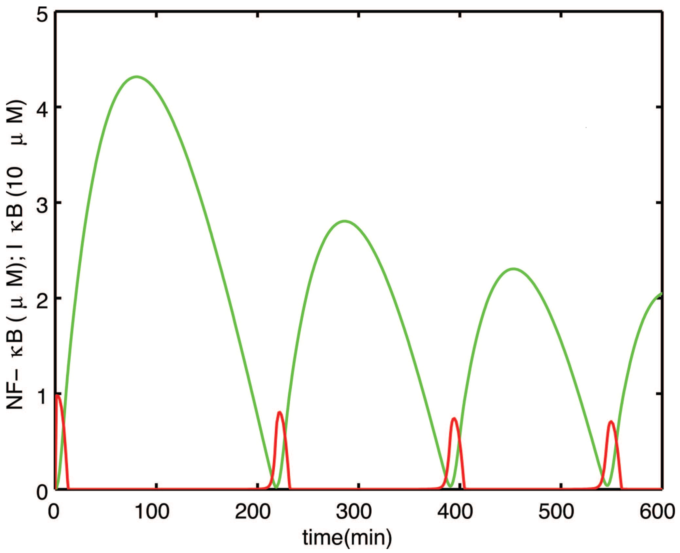

Figure 1: **Krishna *et al.* 2006, Fig. 2** – response to sustained TNF stimulation. Red line – nuclear NF-κB; green line – free cytoplasmic IκBα.

The problem is that these trajectories were obtained for cells with high overexpression of IκBα. As a consequence, once NF-κB enters the nucleus, it triggers synthesis of huge amounts of IκBα that prevents nuclear NF-κB entry for 3 or more hours (Krishna *et al.*, Fig. 2, and Nelson *et al.,* Fig. 3B, left panel). In cells without IκBα overexpression, the oscillation period is much shorter (of about 100 min) and the type of oscillations is different (see Nelson *et al.,* Fig. 2C – cells without IκBα-EGFP; or Hoffmann *et al.* 2002, Figs. 1C and 2C – three different cell lines without IκBα transfection).

The other problem arises, when a pulsatile TNF stimulation is considered. The Authors propose that 'the external signal is supplied by IKK that enters the equations through the parameter C, which is proportional to IKK concentration'. As a consequence, when TNF stimulation terminates, then $C = 0$ implying $\dot{I} \geqslant 0$, which means that after TNF pulse, IκBα may not decrease. For example, if in Figure 1 TNF stimulation had been terminated after 80 min, IκBα would have remained above the observed peak

level, and, as a consequence, a cell would need hours to respond to a following TNF pulse, while Ashall *et al.* 2009 found that cells respond promptly to TNF pulses separated by 100-min long intervals (see Figure 2).

The problem could be possibly solved by adding a new term with spontaneous IκBα degradation. It is possible also to assume that TNF stimulation is represented by a rise of parameter A from 0 to a nominal level, while keeping $C = \text{const}$ (even if this is not in line with the biological mechanism, in which TNF activates IKK, and IKK leads to an increased degradation of IκBα). For $A = 0$, the system possess a well-defined (physiological) steady state (0, 0, 0), which may suggest that Figure 1 was produced by changing A from 0 to the nominal value. However, even under this assumption, we failed to satisfactorily fit the Krishna *et al.* 2006 model to Lipniacki *et al.* 2004 model trajectories: the average multiplicative distance (AMD) was 2.07, while for our reduced model, the fold difference is 1.29. The variable that is not fitted satisfactorily, especially for the pulsatile stimulation, is IκBα protein, which is not dropping in between TNF pulses (see Suplementary Figure S6), which is also in contrast to the Hoffmann *et al.* 2002 model (see Supplementary Figure S1), where the Lipniacki *et al.* 2004 and the Hoffmann *et al.* 2002 models are compared).

(B) The second model mentioned by Referee 3 is the Jensen & Krishna 2012 model. This model supplements Krishna *et al.* 2006 model with two equations governing active and inactive IKK. It is assumed that IKK dynamics depends on A20, but the level of A20 is assumed constant (so A20 does not mediate a negative feedback loop), whereas it is known that A20 is strongly NFκB inducible (see Lee *et al.* 2000, Fig. 2A and Fig. 2D). As in the previous model, the Jensen & Krishna 2012 model has no spontaneous IκBα degradation term, and, as a consequence, in the absence of TNF stimulation, IκBα may be arbitrarily large (however, in important contrast to previous model IKK after TNF pulse decreases exponentially, which allow for the continued IκBα degradation). Finally, the model uses 15 dimensional variables and as such may not be identified based on experiments without absolute measurements of proteins or mRNA levels.

(C) In the third paper mentioned by the Referee 3, Quaiser & Mönnigmann (DOI: 10.1186/1752-0509-3-5) performed identifiability analysis of three regulatory pathways models, including the Lipniacki *et al.* 2004 model, concluding that all three analyzed models are non-identifiable. Considering several reductions steps, the authors stated that the Lipniacki *et al.* model can be simplified to become identifiable be removing A20 (which as said is a mediator of a key negative feedback loop). The final reduced model **is not provided**, the authors state only: "If we also remove A20, the resulting simplified model closely resembles the minimal model proposed by Krishna *et al.*"

Agreement with the experimental data. The original model was developed based on experimental data that include experiments on both WT and A20-deficient cells, as detailed in Lipniacki *et al.* 2004. In the revised manuscript we refer the readers also to data published after 2004 that support both the original and the reduced model. For the Reviewers' and the Editor's convenience we include the comparison figure below.

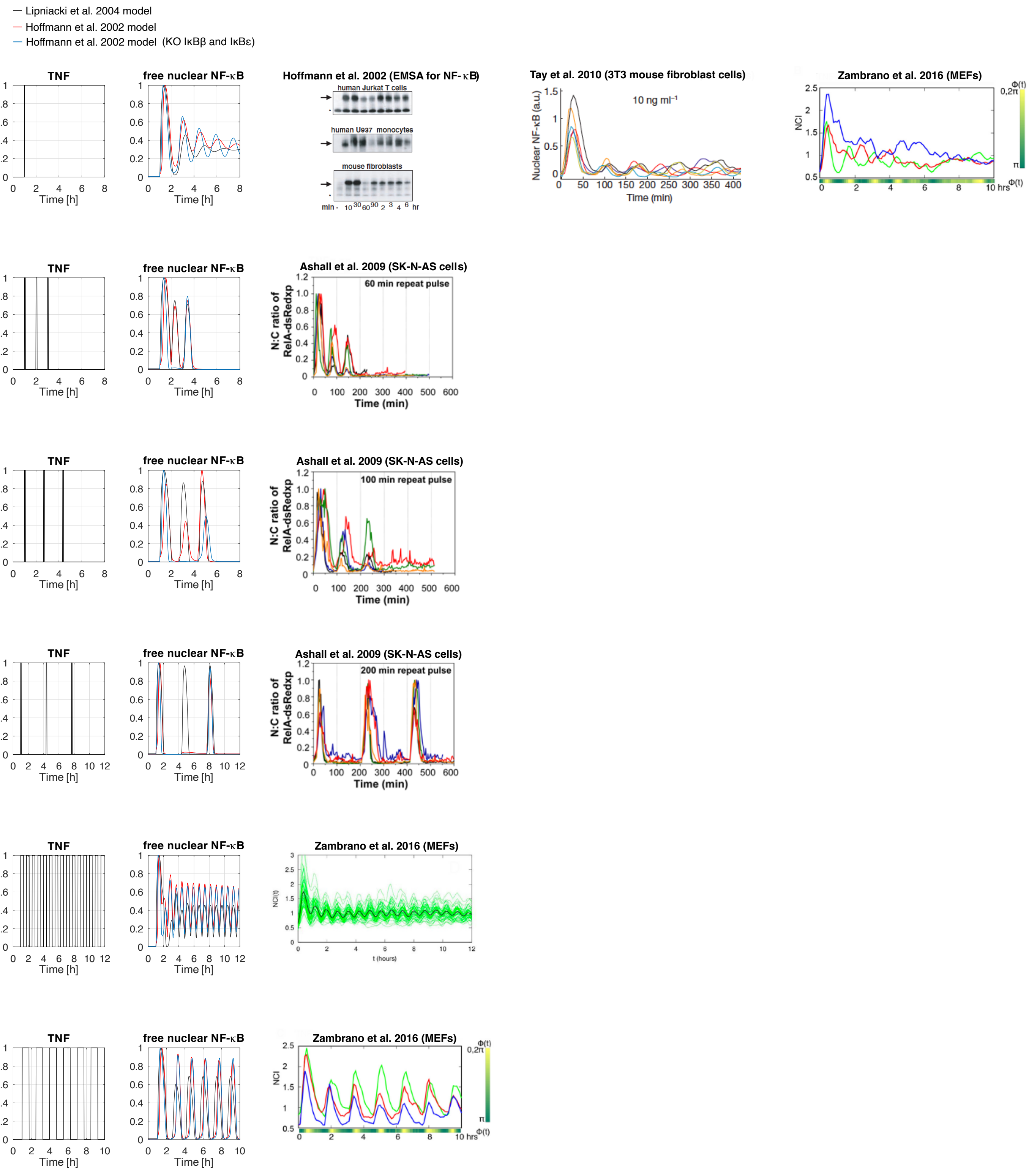

Figure 2: **Comparison of the Lipniacki *et al.* 2004 model and the Hoffman *et al.* 2002 model with experiments performed before and after 2004.**

Response to the report from Reviewer 1

> Reviewer #1: The manuscript entitled "A plausible identifiable model of the canonical NFκB signaling pathway" by Jaruszewicz-Błońska *et al.* reports an identifiable reduced NFκB signaling network. The authors reduce an existing NFκB model from 15 equations to 6; then based on the simulation data, they parameterized the reduced model. Through linear analysis of both the original model and the reduced model (at the set parameter), and Monte Carlo simulation, they claim that the fitted reduced model is identifiable. They also found the reduced network is capable of generating dynamics of adaptation, damped and sustained oscillations. The work does not develop a new approach or method for model reduction, and so only the specific model and its analysis constitute reportable results. While the work is probably technically sound, to this reviewer there are substantial concerns about rationale, approach, and ultimately interest.

Response: The NF-κB pathway is a key pathway that governs the innate immune response and has been implicated in carcinogenesis. We think that the strength of our study is in construction of a reduced, identifiable model of this pathway. The model is supported by experimental data for WT and A20-deficient cells. Although simplified models were proposed (as discussed above), to the best of our knowledge none of them accounted for two negative feedback loops (mediated by IκBα and A20) and for none of them a practical identifiability analysis has been performed.

> **Major points:**
>
> **1.** The motivation/benefits for reducing the model to being identifiable are not fully explained: in the reduced model, parameters generally do not represent physicochemical constants, and cannot be related to diverse biochemical measurements, unlike bigger/more detailed models. Further, numerous, both physico-chemical and reduced models have been previously published of the NFκB regulatory circuit. So the motivation is unclear.

Response: We must agree with the Referee that in reduced models, parameters generally do not represent physicochemical constants, and cannot be related to diverse biochemical measurements. This is a clear disadvantage of reduced models. In detailed models, parameters can represent physicochemical constants, but in turn their values remain undetermined. We think we need both the detailed models and the reduced identifiable models that can be used as building blocks of larger regulatory networks. We refered to this limitation of reduced models in the revised Discussion.

> **2.** The authors reduce the model by 6 steps. it's not justified why they settle on the 6 equations model. Whether could any other network with equation numbers between 6 to 15 be identifiable? If yes, then more equations will reveal more biological details and insights, which could be better than the 6-equation network. Whether could the model be reduced furthermore while keeping identifiability and all the dynamic properties of interest? if yes, then what is the minimal network? Further the proposed model is not related to previously published models in the field. There are not only many other bigger models, but also even more minimal models.

Response: We settled on 6 equations as we found that this is a minimal number of equations capable of accounting of two negative feedback loops that were found important for the NF-$\varkappa$B regulation. We explain it now at the end of section *Original two-feedbacks model and its reduction.* In fact, the most similar model by Jensen & Krishna (2012) has 5 equations; the difference is in the equation for A20 present in our model to account for an A20-mediated feedback loop. We agree with the Referee that more equations will reveal more biological details, but will also require measurements of additional components to identify parameters. In Discussion of the revised manuscript we relate our full and reduced model to other main existing models (including the Hoffmann *et al.* model — see Supplementary Figure S1).

> **3.** The authors used simulation data from a previous model instead of experimental data for parameterizing the reduced model and then analyzed the identifiability of the network based on the simulation-data-based parameterization of the reduced model. Hence it is unclear whether the reduced model is capable of recapitulating experimental data. Several labs have provided experimental data from live cell experiments into the public domain.

Response: The original model was developed based on experimental data that include experiments on both WT and A20-*deficient* cells as detailed in Lipniacki *et al.* (2004). In the revised manuscript we refer readers to data published after 2004, which support both the original and the reduced model. For the Reviewers' and the Editor's convenience we included the comparison figure in this Response (as Figure 2 above).

> **Minor points:**
>
> **1.** For fitting the reduced model, how data timepoints from the original NFB model simulation are chosen is not clearly explained. Why are the fitted time points not evenly distributed? For different species, why are different time points sampled? Why do different types of stimulation use different time points?

Response: The timepoints for fitting are distributed more densely in time intervals in which given variables change more rapidly, which typically means higher sensitivity to parameters. We explained it in the last paragraph of the section *Detailed linear identifiability analysis of the reduced model.*

> **2.** The definition of the sensitivity vector dimension is not explained. The difference between the timepoints number (N) and sensitivity vector dimensions is not explained or justified.

Response: In the revised Methods we explain in more detail a construction of a sensitivity matrix that consists of sensitivity vectors, each consisting of derivatives of normalized data points with respect to a given parameter. The difference between a sensitivity vector dimension and a number of time points is a consequence of normalization, i.e, each time data series is normalized by a respective geometric mean, and thus a series of n measurements of a given variable gives n numbers of which $n-1$ are independent. The normalization is reflected by the assumption that only the relative level of proteins and mRNA are

measured. We explained this in the first paragraph of Methods section. Additionally, in the first paragraph of section *Structural identifiability analysis of the original and reduced model* we wrote “It should be noticed that, because of data normalization, the dimension of sensitivity vectors is smaller than the total number of data points - for example series of three data points gives only two idenpendent numbers”.

> **3.** Line 259: is μ median or mean?

Response: exp(μ) is median, corrected, thank you!

Response to the report from Reviewer 2

> In the paper by Jaruszewicz-Blonska et al, the authors explore the issue of how to make a mathematical model of a biological pathway identifiable, such that the parameters can be determined by experimental data. They focus on their earlier published model of the NF-kB pathway, which contained 15 equations, and then apply a “modeler-driven” approach to reduce it down to 6 equations. This is done through several steps, including nondimensionalization, assessing time scales to replace equations for fast variables with steady-state values, and removing intermediates that are not experimentally measured. Once reduced, they show that the model is now identifiable and that it can reproduce the complex dynamics of the original model. Interestingly, they also consider which experimental protocols are best at identifying the parameters and with what certainty, hinting at some practical guidelines for designing optimal experiments to identify model parameters.
>
> Overall, the work is sound and the authors demonstrate a deep understanding of the NF-kB system. However, the novelty and impact of this work for the field is not clear.

Response: We think that many modelers appreciate the importance of identifiable models. Clearly, structural non-identifiablity can be resolved by reparametrization and nondimensionalization, however, the practical non-identifiablity is harder to resolve, even if general methods are known, see Wieland *et al.* (DOI: 10.1016/j.coisb.2021.03.005). To the best of our knowledge, this is the first study in which an identifiable model of complex dynamic pathway is constructed **and simultanously** its dynamics is shown to closely follow the original model, both for the nominal (Fig. 2 and S1 Fig.) as well as for the randomly chosen parameters (Fig. 3 and S2 Fig.) in response to tonic and pulsatile protocols. It is not difficult to reduce a model to an identifiable one, if the conservation of the dynamics is not the second objective. We appreciate that the Referee notices our effort to provide an experimental protocol that suffices to identify parameters — again, identifiablity depends on experimental protocols, which is frequently neglected in the analysis.

> **Major comments**
>
> **1.** The authors suggest two main areas of impact:
>
> **a.** Their reduced model can "serve as a building block for more comprehensive models" of NFϰB-mediated processes. However, over the last decade many papers have built upon mathematical models of the NF-ϰB pathway that are similar to the original model and have gained insight from these. Thus, it's not clear how much for a barrier this has been.
>
> **b.** Their approach "although may not be automatically generalized, suggests that other regulatory pathways models can be transformed to identifiable while retaining their dynamical features". However, assessing time scales to eliminate short-lived model species is a standard way of reducing models. In many cases this overlaps with the practical situation of not being able to measure these species experimentally, which makes parameters difficult to identify and thus steady-state substitutions are made to eliminate these. Nondimensionalization is also used extensively (although perhaps not as much as it should be). The authors demonstrate excellent and rigorous application of these "modeling best practices" but in the end these are standard methods and so the novelty is not clear.

Response: Yes, there are numerous NF-ϰB models, and reduced models build on them. However, to the best of our knowledge all of them are non-identifiable which **does not** mean that they are not informative. The problem is that the "fitted" non-identifiable model typically contains wrong parameters, and thus it is not clear which of its predictions are true which are not.

> **2.** The idea that some experimental designs are better than others at identifying parameters is an important one that is not often emphasized in these types of studies. Given that the authors are essentially advocating for a bespoke method of model reduction and identifiability, can they articulate guidelines for designing informative experiments that are paired with the guidelines they articulate for model reduction? If they lay these out and perhaps show how they extend (or don't extend) to another pathway, this would increase novelty and define an alternative to the algorithmic methods that have been published and which the authors cite (refs. 7-9).

Response: Thank you for this suggestion. We extended our discussion on the proposed on-off protocol that we found optimal for gaining data sufficient to identify model parameters (see the end of the section *Detailed linear identifiability analysis of the reduced model*). We also discuss how the "experimental" time points are chosen. We think that this approach can be used also for other pathways.

> **Minor Comments:**
>
> Line 200: The division appears to be wrong in the statement "the on-off protocol 914/41 ∼ 43 times". Line 297 (also 302): The authors mean "damped" rather than "dumped".

Response: Corrected, thank you!

Response to the report from Reviewer 3 (Mogens H. Jensen)

> The understanding of the transcription factor NF-kB is extremely important both from a fundamental basic science point of view but also for relations to diseases like diabetes as NF-kB controls probably over 1000 downstream genes. Therefore theoretical studies of the genetic regulatory network involving the NF-kB transcription factor is of extreme importance.
>
> In this paper the authors study a model originally introduced back in 2004 including one of the present authors (TL). The main idea of the paper is to reduce it from 15 variables to 6 variables in a way so the reduced model exhibits dynamics that closely follows that of the original model.
>
> I am a 'big fan' of reduced regulatory models as it gives us a much better way to understand the core mechanisms in the dynamics and as such also gives us a deeper understanding of the underlying 'true' biological dynamics and responses. As such I find this paper adds to the understand of this important genetic system. I must however declare a disagreement with one of the first the sentences in the beginning of the introduction, "In this study we reduce the original 15- variable NF-kB model developed by Lipniacki et al in a way that it preserves the dynamics of key variables (in contrast to earlier simplified models [5],[6],[7])." No, exactly the same approaches were applied for the reduction in these papers, and indeed the dynamics of the key variable was conserved and in fact in excellent agreement with experimental data series. Also, nondimensionalization were applied in these references.

Response: In Discussion of the revised manuscript, prompted by the Editor, we discuss more precisely differences between our reduced model and earlier reduced models. We review these differences in more detail in our response to the Editor's comments above. In Introduction we replace the questioned sentence by less controvercial and precise one: "We reduce the original 15-variable NF-κB model developed by Lipniacki et al. in a way that it preserves two negative feedback loops controlling NF-κB activity and mediated by IκBα and A20 (in contrast to ealier even simpler models Krishna et al. 2006, Jensen & Krishna 2012), but makes the model structurally and practically identifiable".

> As far as I can judge the mathematical reduction presented here is ok. Not that I necessarily am able to follow all the included steps but it seems to me sound.
>
> Authors mention NF-kB responses to pulsatile TNF stimulation and refer to refs [16],[17]. However, this was also studied in a lot of details i Ref. [19] (Kellogg, Tay) and in Heltberg *et al*,

> *Cell Systems* 3, 532-539 (2016).

Response: In the revised manuscript we refer to these studies in the context of pulsatile TNF stimulation. We noticed that, in contrast to the studies by Ashall *et al.* (2009) and Zambrano *et al.* (2016), TNF concentration changes in a continuous manner.

> Coming to the results as presented in Figs.2,3 I am a little confused. In the top panel there is a constant TNF addition. However, the oscillations are more or less absent, only 1-2 peaks appear. Do we not expect oscillation over much longer time, actually almost sustained oscillations?

Response: The question is interesting and difficult. We think, based on single-cell data, that in some cell types there are almost sustained oscillations observed at the single-cell level. These oscillations appear damped at the cell population level, just because over time cells become less and less synchronous, so the oscillations "cancel out" (we discussed this in detail in Lipniacki *et al.,* 2006, see Fig. 11, DOI: 10.1529/biophysj.104.056754). We think however that single-cell oscillations are sustained because of the molecular noise associated with gene activation (and, at low TNF doses, TNF receptor activation). As there are two gene copies of the key inhibitor, IκBα, stochastic activation of a single IκBα gene copy (by binding of NF-κB molecule) kicks system out of equilibrium. Our claim is supported (but not proved) by observation that the stochastic version of the deterministic model not exhibiting sustained oscillations (like the Lipniacki *et al.* 2004 model) exhibits oscillations (as in Lipniacki *et al.*, 2006).

> In the other panels the authors are adding pulsatile TNF but only with three pulses. Then, they observe nice entrainment as for instance seen in the two papers mentioned above. Is this correctly understood? Fig.3 is somewhat difficult for me to overview - could it be made more clear?

Response: Yes, our reduced model can be entrained to the pulsatile TNF stimulations (such as shown in Fig. 2), and oscillate as long as such stimulation is provided (see Supplementary Figure S1, we add this comment in the revised text). In fact, Kellogg & Tay, 2015 used a stochastic version of the Lipniacki *et al.* model (developed for our joint Tay *et al.* 2010 *Nature* paper). In order to make the Fig. 3 clearer we expanded its caption.

> Actually, the authors come back in Fig. 8 to sustained oscillations with tonic TNF stimulation which I presume means continuous stimulation with pulses. Indeed limit cycles are observed and therefore I wonder why the authors do not study this in the entrainment sense, that is how the external TNF period couples to the internal NF-kB oscillation. And here there is an important issue as pointed out by Zambrano *et al.* (Ref. [17]) namely that when TNF has pulses up to a level and then down to zero, then one might jump in and out of the Hopf bifurcation (Hopf bifurcation occurs at a specific level of TNF, thus if it pulses above and below it might be a tricky situation).

Response: No, in Fig. 8 the tonic (constant) TNF stimulation is applied (no TNF pulses), and the limit-cycle oscillations are observed, because for panels B and C the parameters are different than the nominal (obtained by fitting the reduced model to the original one).

> I also miss a discussion on how the results of the presented reduced model compare to previously published reduced models? Do they agree, my guess would be they actually do.

Response: We discussed this in the revised Discussion, and also provided a detailed discussion in the response to the Editor's comments. The main, and we think important difference is that other reduced models do not contain A20 negative feedback loop - even if A20 is present like in Jensen and Krishna 2012 model (used also in Heltberg *et al.* study) it is assumed to be constant. We think that more detailed modeling of A20 is important because this protein also inhibits TBK1 - kinase that activates IRF3. NF-κB and IRF3 are jointly activated upon detection of viral RNA and trigger synthesis of inteferons that warn bystander cells about infection. Threfore A20 is implicated in NF-κB and IRF3 crosstalk, see Czerkies et al. 2018 DOI: , Fig. 6.

> There are some corrections needed in the reference list (how authors names come in etc).

Response: Corrected, thank you!

> In conclusion, I as mentioned support approaches based on reduced models. In this paper another attempt is made to include six variables and around 15 parameters. I am positive to the paper but should like the authors to consider all the points I have made above.

Response: Thank you for careful reading, comments, and positive recommendation.

□
