# Supplementary material for: A plausible identifiable model of the canonical NF-κB signaling pathway
Source: PLoS One. 2023 Jun 2;18(6):e0286416. doi: 10.1371/journal.pone.0286416 (PMC10237389; doi:10.1371/journal.pone.0286416)
Supplement: Attachment — Submitted filename: Jaruszewicz_et_al__Response_to_Reviewers.pdf [file pone.0286416.s017.pdf]

Tomasz Lipniacki
Department of Biosystems and Soft Matter
Institute of Fundamental Technological Research
Polish Academy of Sciences

Pawińskiego 5B, 02-106 Warsaw, Poland
Phone: +48 22 8261280
E-mail: tlipnia@ippt.pan.pl
Website: https://pmbm.ippt.pan.pl

Subject: **Resubmission of the revised manuscript PONE-S-23-08732** May 11, 2023

Dear Editor, Dear Reviewers,

Thank you for your thorough reading and comments on our manuscript "A plausible identifiable model of the canonical NF-ϰB signaling pathway". In this document, we provide a concise description of the amendments (directly below) and detailed point-by-point responses to your comments (on following pages).

The main amendment is the following:

In response to the Reviewer #3 and Editor's request we provide a comparison of Lipniacki et al. 2004 model with two models by Ashall et al., 2009 and Murakawa et al., 2015, discussed in Mothes et al., Frontiers Physiology 2020 11, 896, that share a common core pathway module with Lipniacki et al. 2004 model, but differ in A20 feedback structures. Supplementing the extensive analysis and discussion of these three models by Mothes et al., we compared responses of these models to the tonic as well as five pulsatile TNF stimulation protocols (proposed earlier by other researchers and used in our identifiability analysis). We considered responses of both wild type cells, S2 Fig, and A20 deficient cells, S3 Fig (the latter were neither analyzed by Mothes et al., 2020, nor in the original papers). In addition, in response to the former Reviewers requests, we compared our model with the first comprehensive model by Hoffmann et al., 2002 (that omitted A20 feedback loop), see S1 Fig. The reduced model developed in the current paper is also compared with the first minimal NF-ϰB model developed by Krishna et al. , 2006 in S8 Fig. The mathematical details of all four reviewed models (by Hoffmann et al., Ashall et al., Murakawa et al. and Krishna et al.) are provided in S1 Text; numerical codes written to execute these models are provided as S1 Codes. We think that with all these comparisons our results are adequately placed in the existing computational analysis of NF-ϰB system.

A point-by-point response to the Reviewers' comments is provided below.

We hope that you will find the revised manuscript suitable for publication in *PLOS ONE*.

Sincerely,

Tomasz Lipniacki
Professor and Head

Response to the report from Reviewer 1

> Reviewer #1: In this paper the authors reduce an existing model of the canonical NF-κB pathway from 15 to 6 equations The reduced model retains two negative feedback loops mediated by IκBα and A20. A sensitivity-based linear analysis and a Monte Carlo-based analysis demonstrates that the resulting model is both structurally and practically identifiable given measurements of 5 model variables from a simple TNF stimulation protocol. The reduced model is capable of reproducing different types of responses that are characteristic of regulatory motifs controlled by negative feedback loops including nearly-perfect adaptation as well as damped and sustained oscillations. The paper is well considered, well written and the authors offer excellent evidence and analyses that they have reduced the original NF-κB pathway model to a model that is identifiable, but still capable of reproducing the rich dynamics of the original model in response to tonic as well as pulsatile TNF stimulation. The approach and new model makes a significant contribution to the field of Systems Biology.

Response: We are grateful for positive assessment of our study.

Response to the report from Reviewer 2

> In this interesting work Jaruszewicz-Blonska et al. propose a new mathematical model of the canonical NF-κB system by drastically reducing the dimensions of a previous existing model. After this, they perform a number of numerical simulations to assess the 'identifiability' of the model. In particular they show that different combinations of simulated data from the original model lead to a better 'identifiability' when fitted with the reduced model than with the original one, while being able to reproduce the dynamics of the original model with reasonable accuracy. Very importantly, they are able to identify potential "experimental protocols" that would produce data that would make the reduced model identifiable. The reduced model is also identifiable for in-silico data blurred by noise. Finally, it is shown that the reduced model displays the qualitative dynamics observed in different experimental contexts, including damped and persistent oscillations.
>
> Although model reduction should in principle contribute to a better identifiability of the parameters, to my knowledge this is the first time in which this has been systematically explored for NF-κB signaling models, and the fact that the proposed model is more identifiable for this in-silico data carries important insights to be considered when opting between simple or complicated models of a signaling system. I also found of particular interest the fact that they show how specific "experimental" protocols might lead to better identifiability. The paper is well written and in my opinion technically very sound.

Response: We are grateful for favorable assessment of our study.

> **1.** My main (yet minor) concern is related with the way authors use the concept of "identifiability". In my opinion, identifiability refers always to certain kind of data to which the model shall be fit, and what we can say for their reduced model is that the model is identifiable when trying to fit in-silico data produced by the original model. This is something that, I underline again, is of theoretical interest by itself and potentially useful for applications, e.g. to fitting to real experimental data, something that probably is beyond the scope of the manuscript. But I think that this should be stated more explicitly. This is an issue that is somehow indicated by the previous reviewers of the manuscript (I thank the authors for including these reports): Reviewer 1, raises in point 3 how "it is unclear that the reduced model is capable of recapitulating experimental data". The authors reply saying that their reduced model can qualitatively reproduce the original model, which itself was "based on experimental data". However, it is unclear to me if the original model (and hence the reduced one) was able to qualitatively or quantitatively (with a small fitting error) reproduce experimental data. The figure shown indicates the former, not the latter. It could well be that when trying to fit experimental data to the reduced model, the discrepancy is high and some parameters might not be identifiable. Hence I'd suggest that the authors discuss this in more detail and to specify more clearly (maybe also in the title) that the reduced model is identifiable with respect to in-silico data generated from the higher dimensional original model. I think that this way their interesting contribution will become more clear.

Response: The linear identifiablity analysis is performed by perturbing parameters with respect to their nominal values, either of the original model or reduced model fitted to original. The Monte Carlo-based practical identifiablity analysis is performed based on in-silico generated measurements (by the reduced model) as detailed in section *Practical identifiability analysis of the reduced model based on Monte Carlo simulations.* The Referee is right in saying that for different parameters obtained from fitting to some experimental data some parameters might not be identifiable. Respectively, at the end of the section we wrote: *The performed linear as well Monte Carlo indentifiability analyses are local in that sense that they start from the nominal parameter values. One can expect that for substantially different parameters the reduced model can be found non-identifiable.*

> A second related minor concern is that the reduced model relies on approximations on the original model that in some cases are based on the existence of "fast" processes. However I speculate that the ability to identify the parameters and closely reproduce the original model with high accuracy might be limited to ranges of the original parameter set for which these reactions can still be considered "fast". This might not need to be tested with additional simulations, but I'd like to see this discussed in the manuscript. Overall I think that this is an interesting manuscript that might require small corrections to put on a clearer light the contribution to the NF-κB modeling field made by the authors.

Response: We mentioned this limitation discussing Fig. 3 and S5 Fig in which we show that the reduced model after refitting is able to reproduce the original model, even if the parameters of the original model are randomly varied 3-fold, which results in markedly different dynamics.

Response to the report from Reviewer 3

> The manuscript entitled "A plausible identifiable model of the canonical NF-κB signaling pathway" by Jaruszewicz-Blonska et al. already went through a major revision. While I was not part of the review process I agree with the three reviewers that the work described in the manuscript is 'probably technically sound', but there 'substantial concerns about the approach and ultimate interest'. While I find the questions of the reviewers highly relevant these are not fully answered by the authors. The main strength put forward by the authors that their study constructs a reduced, identifiable model and 'to the best of their knowledge the first accounting for two negative feedback loops (mediated by IκBα and A20)' is not correct. Mathematical models for the NF-κB system have been recently reviewed in a paper of Haga and Okada, Biochemical Journal 2022, 279. 161 who report on several NF-κB models including the A20 feedback beside the IκBα feedback. Highly relevant for the approach introduced here seems the paper of Mothes et al. Frontiers Physiology 2020 11, 896 that determined the impact of different A20 implementations on the dynamics of NF-κB in models of a comparable size as the one developed here. A comparison with the other minimal models should include these models as well.

Response: We thank the reviewer for pointing out the paper by Haga and Okada, Biochemical Journal 2022, 279. 161. We have included this reference in the manuscript. The paper of Mothes et al., 2020 discusses the impact of different implementations of A20 feedback on NF-κB dynamics comparing the original Lipniacki et al., 2004 model with two latter models by Ashall et al., 2009 and Murakawa et al., 2015. Importantly, Murakawa et al. model consists of 7 equations, thus only one more than the proposed reduced model. Supplementing in-depth discussion in Mothes et al., 2020, we compared responses of these three models to tonic as well pulsatile TNF stimulation, both for wild type (WT) cells, S2 Fig and A20 deficient cells, S3 Fig (the latter were neither analyzed by Mothes et al., 2020, nor in the original papers). In brief, the Lipniacki et al. 2004 model assumes that upon TNF stimulation IKK transits from its resting form, IKKn, to active form IKKa. The A20 protein, in turn, promotes transition of IKKa to inactive form, IKKi. Consequently, in WT cells in response to the tonic stimulation, IKKa exhibits a short pulse with a low tail (S2 Fig), while in A20-deficient cells, the tail is higher, because the transition from IKKa to IKKi proceeds at a lower rate (S3 Fig). Ashall et al. assumed also existence of these three forms of IKK, but proposed that A20 instead of promoting IKKa to IKKi transition, slows down the transition from IKKi to IKKn. The consequence of this assumption is similar: in WT cells, IKKn is more depleted than in A20-deficient cells, and consequently the tail of IKKa is lower (compare S2 Fig and S3 Fig). These two models produce IKKa profiles in agreement with the experiment conducted by Lee et al., 2000), that motivated incorporation of A20 feedback to NF-κB modeling. These experimental data (Lee et al., 2000, see Fig. 3E therein) indicate a pronounced peak of IKKa 10 minutes after the beginning of TNF stimulation followed by a tail of IKK activity - low for WT cells and higher for A20-deficient cells. A substantially different IKK-A20 module structure was proposed in the Murakawa et al. 2015 model,

which accounts only for one (active) form of IKK. Accumulation of active IKK is slowed down by A20, while the depletion term of active IKK is constant. Consequently, in WT cells, due to accumulation of A20, active IKK also exhibit a peak (S2 Fig). However, in A20-deficient cells, the level of active IKK grows monotonically to some asymptotic value (S3 Fig), which is in stark contrast to Lee et al., 2000 data. For WT cells, the Murakawa et al. 2015 model (as well as the Hoffmann et al. 2002 model) fails to respond to 3 TNF pulses separated by 200 min intervals (S1 Fig, S2 Fig) that as demonstrated by Ashall et al. 2009 induce 3 peaks of NF-κB activity; this discrepancy is possibly a consequence of models parametrization not A20-IKK module structure, or lack of this module. We included the above text in the second paragraph of the Results section.

> Also the question (reviewers 1 major points) whether the reduced model can recapitulate experimental data (e.g. from literature) is not convincingly addressed.

Response: The reduced model (as shown in Fig. 2) closely follows the original Lipniacki et al. 2004 model that was calibrated based on the existing literature on WT and A20-deficient cells, but also reproduces latter experiments of Ashall et al., 2009 and Zambrano et al., 2016., as discussed in the beginning of the Results section referring to S1 Fig.

> In that context I find the argumentation of the authors to reviewer 2 with respect to 'non-identifiable models' as 'typically containing wrong parameters', arguing 'thus it is not clear which of the predictions are true and which not' contradictory to the approach to fit the model to the simulated dynamics of exactly such 'non-identifiable models'- this seems to be a contradictory approach that needs clarification.

Response: We do not see contradiction. After fitting a non-identifiable model the researcher still does not know true parameter values, and therefore *a priori* may not be certain about model predictions, especially for variables that were absent in data set used for model fitting. For example, if only a certain protein level is measured, typically a model will not be able to predict correctly the corresponding mRNA level.

□
